# Microbiome Composition and Function in Aquatic Vertebrates: Small Organisms Making Big Impacts on Aquatic Animal Health

**DOI:** 10.3389/fmicb.2021.567408

**Published:** 2021-03-11

**Authors:** Ludek Sehnal, Elizabeth Brammer-Robbins, Alexis M. Wormington, Ludek Blaha, Joe Bisesi, Iske Larkin, Christopher J. Martyniuk, Marie Simonin, Ondrej Adamovsky

**Affiliations:** ^1^RECETOX, Faculty of Science, Masaryk University, Brno, Czechia; ^2^Department of Large Animal Clinical Sciences, University of Florida, Gainesville, FL, United States; ^3^Department of Physiological Sciences, University of Florida, Gainesville, FL, United States; ^4^Center for Environmental and Human Toxicology, University of Florida, Gainesville, FL, United States; ^5^Department of Environmental and Global Health, University of Florida, Gainesville, FL, United States; ^6^Univ Angers, Institut Agro, INRAE, IRHS, SFR QUASAV, Angers, France

**Keywords:** microbiome, fish, aquatic mammals, stressors, biomonitoring, ecosystem health

## Abstract

Aquatic ecosystems are under increasing stress from global anthropogenic and natural changes, including climate change, eutrophication, ocean acidification, and pollution. In this critical review, we synthesize research on the microbiota of aquatic vertebrates and discuss the impact of emerging stressors on aquatic microbial communities using two case studies, that of toxic cyanobacteria and microplastics. Most studies to date are focused on host-associated microbiomes of individual organisms, however, few studies take an integrative approach to examine aquatic vertebrate microbiomes by considering both host-associated and free-living microbiota within an ecosystem. We highlight what is known about microbiota in aquatic ecosystems, with a focus on the interface between water, fish, and marine mammals. Though microbiomes in water vary with geography, temperature, depth, and other factors, core microbial functions such as primary production, nitrogen cycling, and nutrient metabolism are often conserved across aquatic environments. We outline knowledge on the composition and function of tissue-specific microbiomes in fish and marine mammals and discuss the environmental factors influencing their structure. The microbiota of aquatic mammals and fish are highly unique to species and a delicate balance between respiratory, skin, and gastrointestinal microbiota exists within the host. In aquatic vertebrates, water conditions and ecological niche are driving factors behind microbial composition and function. We also generate a comprehensive catalog of marine mammal and fish microbial genera, revealing commonalities in composition and function among aquatic species, and discuss the potential use of microbiomes as indicators of health and ecological status of aquatic ecosystems. We also discuss the importance of a focus on the functional relevance of microbial communities in relation to organism physiology and their ability to overcome stressors related to global change. Understanding the dynamic relationship between aquatic microbiota and the animals they colonize is critical for monitoring water quality and population health.

## Introduction

Aquatic ecosystems are under stress from global change due to both anthropogenic and natural phenomena. Climate change, ocean acidification, eutrophication, and pollution have affected microbial and host dynamics in aquatic animals. Environmental factors such as light, temperature, and oxygen affect the smallest members of our freshwater and oceanic habitats. There is a close and delicate interaction between microbial communities and animals, and perturbations of this kind of symbiotic relationship can lead to ecological disruptions. These impacts on microbiota can be significant and may influence the health of aquatic vertebrates.

In this review, we discuss the current knowledge and highlight the importance of interactions between microbiomes, environments, and hosts, discussing the role of microbiomes in relation to freshwater and marine fish and mammals. We recognize that aquatic invertebrates comprise a significant biological group whose microbiomes are essential for aquatic food webs, however, here we focus on larger freshwater and marine vertebrates because many are vulnerable to global climate change and pollution. First, we review the functional role of planktonic microbiomes in water and their influence on tissue microbiomes of aquatic animals. Second, we review what is known about the composition and function of gut, skin, and gill microbiomes in fish, as well as ecological drivers behind tissue microbiome assembly. Third, we discuss microbiome research in relation to marine mammal conservation. Having reviewed literature concerning microbiome in association with aquatic vertebrates, we discuss how their symbiotic relationships may be influenced by environmental stressors as case studies on emerging contaminants of two categories. This is followed by case studies of emerging contaminants, and we select cyanobacteria and micro-plastics to highlight in this review; however, we point out that there are various examples that can be discussed in the context of environmental stressors (e.g., chemicals, abiotic factors). We complete the review with a discussion of the potential for using microbiome measurements as an indicator of aquatic ecosystem health and suggest future directions that can advance the science.

## The Microbiome of Aquatic Habitats

Many of the inert components of water are composed of inorganic elements and molecules, however, water can almost be considered a living matrix due to its numerous biological inhabitants. The microbes (archaea, bacteria, fungi, protists) residing in water perform numerous biological functions, but also serve as a source for the microbiomes of host animals, including invertebrates, fish, and aquatic mammals (Krotman et al., 2020). Interest in the microbiology of water spans centuries, as early scientists recognized the ability of microbes in the water to influence the health of animals and humans. These early examples include the recognition of water as a vector for numerous infectious microbes, with examples including *Campylobacter jejuni*, toxigenic *Escherichia coli*, *Shigella* sp., *Vibrio cholerae*, and *Salmonella enterica* (Ashbolt, 2004). Perhaps now more than ever, there has been a renewed focus on understanding how microbiomes in drinking water influence our resident microbiomes (Pinto et al., 2012, 2014; Ji et al., 2015; Proctor and Hammes, 2015; Ling et al., 2018). In addition to interacting with host organisms, free-living microbes have an essential role in aquatic ecological processes.

Microbes perform a number of important ecosystem services in water. Perhaps the greatest function that microbes are involved with is the primary production of energy from CO_2_. While much of this is done by photosynthetic plants, algae, and cyanobacteria, there are also species of bacteria that can chemosynthesis the backbones of important biological molecules including lipids, proteins, complex carbohydrates, and nucleic acids (Sokoronin, 1966; Dubilier et al., 2008). Chemosynthesis was originally thought to be performed only by extremophilic bacteria living in deep ocean areas where light does not reach; however, the discovery of proteorhodopsin, a gene allowing bacteria to harvest energy from sunlight without photosynthetic machinery, was a breakthrough in recognizing the importance of microbes in primary production (Moran, 2015). Microbes also support ecosystems through their involvement in nutrient cycling, especially the nitrogen cycle (McKenney et al., 2018). Interestingly, a study of ∼140 samples of ocean water from around the world revealed that 73% of the prokaryotic gene abundance in all of the ocean samples can be attributed to the same functional core of the human gastrointestinal microbiome. However, there were also key differences in functions between the systems; the human microbiome prioritizes immune defense, signal transduction, and metabolism, while the ocean microbiome prioritizes general transport mechanisms of important biomolecules (lipids, nucleotides, amino acids) and energy production (Sunagawa et al., 2015). While the above are merely examples of the many services that the water microbiome performs, it is clear that these are essential services supporting all life in water.

The composition of the water microbiome can vary widely geographically, temporally, and seasonally, however, there is a demarcation for a core microbiome whose membership is predictable over seasons, ocean depths, and organic matter features (Moran, 2015). An environmental driver with the greatest influence on the water microbiome is temperature (Sunagawa et al., 2015), especially when compared to other factors like geography and water depth. A notable example is the influence of temperature on the growth of toxigenic freshwater cyanobacteria. Numerous studies have indicated that there are threshold water temperatures required for cyanobacterial blooms (Lehman et al., 2013; Mowe et al., 2017; Wood et al., 2017), an important concern in light of global climate change. While changes in global water temperatures are one of the largest concerns for the maintenance of microbiome function in water, rising levels of carbon dioxide, the major driving factor of global climate change, may have a greater influence on aquatic microbes than temperature. Minich et al. (2018) found that between the addition of climate change stressors (rising temperature and CO_2_), rising CO_2_ had a greater impact on seawater microbiomes than temperature. While research has shown that the composition of a water microbiome is dynamic with changing temperature, seasons, geography, and other water quality parameters, the relationship between water microbiomes and aquatic organisms is not extensively studied.

Aquatic animals are surrounded by a milieu of microbes, and evidence suggests that the composition of environmental microbiomes influences the microbiomes of aquatic hosts. For example, in zooplankton, alterations of the environmental microbiome by anthropogenically introduced antibiotics causes a shift in the host-associated microbiome, which in turn influences growth of plankton populations (Callens et al., 2018). Additional studies also indicate that newborn and juvenile invertebrates and fish raised in abiotic environments resist the development of a host microbiome, which typically has consequences for growth and survival (Rawls et al., 2004; Sison-Mangus et al., 2015). However, research indicates that the surrounding water microbiome does not always mirror the microbiome of aquatic hosts. For example, a study by Krotman et al. (2020) comparing microbiomes of a heterogenous freshwater system and the skin microbiomes of numerous freshwater fish revealed the ability of the skin to concentrate beneficial microbes, even when surrounded by a homogenous mixture of microbes. A meta-analysis of the gut microbiomes of freshwater, estuarine, and saltwater fish and surrounding environmental samples indicated that the gut microbiome is not a reflection of the local habitat but rather the specific gut environment of the fish and local selective pressures (Wong and Rawls, 2012).

In summary, the water microbiome plays an important role in the ecology of both freshwater and marine systems, performing essential ecosystem services and providing the energy for these systems. The composition of the water microbiome is dynamic, with variable composition over time, geography, and environmental conditions. However, the various functions of the water microbiome are conserved globally, suggesting core microbial functions in water. The microbiomes of animals inhabiting water are influenced by the composition of their local water microbiome, as the water microbiome serves as a reservoir for the microbes that comprising the microbiome of aquatic animals. However, aquatic animal microbiomes are not a direct reflection of the surrounding water microbiome, as the host-associated microbiome is under numerous environmental and biological selection pressures. In the following sections, we will examine the diversity of aquatic vertebrate microbiomes and their important functions within the hosts.

## Fish-Associated Microbiomes

**FIGURE 1:**
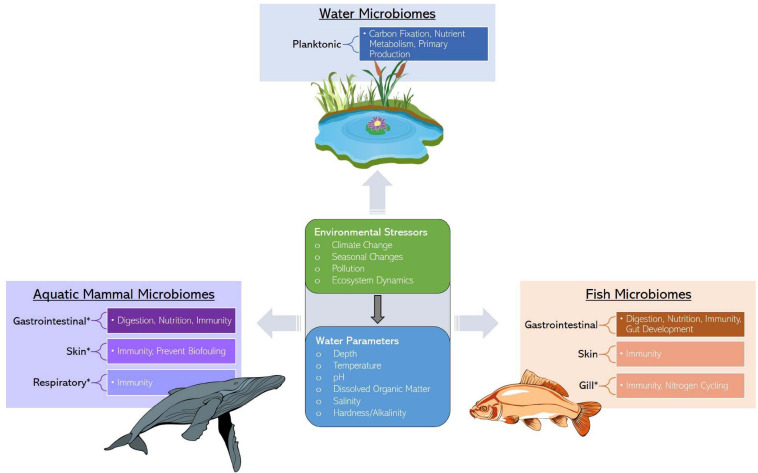
Services provided by water, marine mammal, and fish microbiomes along with the ecological stressors and factors influencing microbiome assembly. Asterisks represent an identified research gap.

Fish and their tissue microbiomes have co-evolved over time in an aquatic milieu of microorganisms, establishing mutually beneficial relationships. Fish microbiomes are involved in the host’s biological functions (e.g., nutrient acquisition and immunity, including competitive suppression of pathogens) and in return the host supports the nutrition pool and colonization of both internal and external microbiota. This section summarizes the most recent findings and describes the mutualism between microbiomes and fish, specifically emphasizing the role of less-studied skin and respiratory microbiomes in addition to the intestinal microbiome. We synthesize the available knowledge on the importance, composition, and environmental factors influencing the structure of fish core microbiomes (Figure 1). Additionally, we include a table describing the proposed functional roles of microbial taxa in fish species and tissues (Table 1 and Supplementary Table 1). In the subsequent section (see section “Marine Mammal-Associated Microbiome”), we present in a similar fashion what is known about marine mammal microbiomes in different tissues, to facilitate comparisons among aquatic vertebrates.

**TABLE 1:** The summary of the microbial orders and their organ specific function in different fish species.

Tissue	Function	Microorganisms (Order)	Fish	Habitat	References
Gills	Denitrification	Nitrosomonadales	Carp, zebrafish	Freshwater	Hess et al., 2015
Gills	Immunity and Stress Response	Alteromonadales, Bacillales, Burkholderiales, Campylobacterales, Corynebacteriales, Cytophagales, Desulfobacterales, Enterobacterales, Ferrovales, Flavobacteriales, Micrococcales, Oceanospirillales, Parachlamydiales, Pelagibacterales, Propionibacteriales, Pseudomonadales, Rhodobacterales, Salinisphaerales, Sphingomonadales, Thiotrichales, Vibrionales	Kingfish, salmon, clownfish	Saltwater	Steinum et al., 2009; Lowrey et al., 2015; Legrand et al., 2018
Intestine/Feces	Diet and Nutrient Metabolism	Saccharomycetales, Aeromondales, Alteromonadales, Bacillus, Clostridiales, Corynebacteriales, Enterobacterales, Flavobacteriales, Fusobacteriales Micrococcales, Pseudomonadales, Rhizobiales, Vibrionales	Carp, catfish, tilapia, wrasse, puffer, surgeonfish	Freshwater, Saltwater	Smriga et al., 2010; Xing et al., 2013; Ni et al., 2014
Intestine/Feces	Immunity and Stress Response	Bacillus, Bacteroidales, Brevinematales, Burkholderiales, Chitinophagales, Clostridiales, Flavobacteriales, Fusobacteriales, Mycoplasmatales, Rhizobiales, Rhodocyclales, Veillonellales, Vibrionales	Zebrafish, trout, turbot	Freshwater, Estuarine	Smriga et al., 2010; Falcinelli et al., 2015; van Kessel et al., 2016
Intestine/Feces	Water Temperature Response	Vibrionales	Salmon	Saltwater	Hanning and Diaz-Sanchez, 2015
Skin	Hydrochemical Adaptation	Rhizobiales, Rhodospirillales, Vibrionales	Piranha, cichlid	Freshwater, saltwater	Wilson et al., 2008
Skin	Immunity and Stress Response	Alteromonadales, Bacillales, Burkholderiales, Campylobacterales, Corynebacteriales, Ferrovales, Micrococcales, Oceanospirillales, Pseudomonadales, Rhodobacterales, Rhodocyclales, Sphingomonadales, Synechococcales, Vibrionales, Xanthomonadales	Kingfish, clownfish, kingfish, charr, trout	Freshwater, Saltwater	Schmidt et al., 2015; Brown et al., 2018; Chiarello et al., 2018; Legrand et al., 2018
Whole Body	Salinity Response	Aeromondales, Alteromonadales, Fusobacteriales, Vibrionales	Mexican molly	Freshwater, Estuarine	Durack et al., 2012

*The detailed information about microbial genera and insight into their specific function is presented in Supplementary Table 1.*

### Respiratory Microbiome

Gill surfaces are the primary site of gas exchange in fish and are colonized by microbial communities. Like the skin and gut, the gills are coated in a layer of mucus that is constantly replaced, providing both a defense against and habitat for pathogenic or commensal prokaryotes (Lumsden et al., 1995; Gomez et al., 2013). Antimicrobial peptides found in the skin and intestines of fish are also present in the gill mucus, highlighting the importance of the gills as a first line of defense against pathogenic infection (Iijima et al., 2003; Murray et al., 2003). Gill microbiota can reflect internal and external diseases (Legrand et al., 2018). For example, farmed Atlantic salmon (*Salmo salar*) with proliferative gill inflammation had different gill microbiomes than healthy fish, with a lower abundance of important Gammaproteobacteria (*Psychrobacter*) and a higher abundance of potentially pathogenic bacteria, such as *Tenacibaculum* and *Flavobacterium* (Steinum et al., 2009).

Gills of reef fish are dominated by class Gammaproteobacteria but also enriched in Betaproteobacteria and Alphaproteobacteria compared to other tissues (Murray et al., 2003; Reverter et al., 2017; Brown et al., 2018). Host-specific characteristics such as life stage, species, and diet influence the composition and diversity of the gill microbiome. A study sequenced the gill microbiomes of 53 species of reef fish in French Polynesia and found significantly different compositions between adult and juveniles — though the authors cautioned that this association was confounded by fish species, which was not controlled for due to highly variable sample sizes for each family. Adult fish categorized by diet had slightly different gill microbiome compositions, with carnivores clustering more tightly together. Again, comparisons were confounded by fish species (Pratte et al., 2018). The pairwise similarity coefficient in gill microbiomes of gibel carp (*Carassius auratus gibelio*) and bluntnose black bream (*Megalobrama amblycephala*) was marginally different (Wang et al., 2010). Sequenced gill microbiomes of four species of butterfly fish shared only 26 of 1041 total OTUs. Gills were dominated by Proteobacteria for all species, but the proportions of Alpha, Beta, and Gammaproteobacteria as well as genera composition fluctuated between species (Reverter et al., 2017). Notably, the gills of two butterfly fish species had a high proportion of *Vibrio* (class Gammaproteobacteria), which is in agreement with a study that found high levels of *Vibrio* in the gills of wild-caught red snapper (*Lutjanus campechanus*) (Tarnecki et al., 2016).

As fish excrete ammonia and other nitrogenous compounds through the gills, it is theorized that nitrogen-fixing bacteria may be enriched in the gill mucus. Indeed, Legrand and colleagues reported enrichment of *Nitrosomonas*, chemoautotrophic bacteria that convert ammonia to nitrite, in the gills of yellowtail kingfish (*Seriola lalandi*) (Legrand et al., 2018). Wild-caught red snapper also displayed elevated levels of *Nitrosomonas* in the gills (Tarnecki et al., 2016). In another study, van Kessel et al. (2016) reported genes for ammonia-oxidizing and denitrifying and bacteria in the gills of common carp (*Cyprinus carpio*) and zebrafish (*Danio rerio*), and showed that these bacteria convert ammonia to nitrogen gas.

Gill microbiomes respond to changes in the environment. Following exposure to suspended sediments, clownfish (*Amphiprion percula*) larvae gill microbes had higher abundances of pathogenic taxa (*Flavobacterium*, *Pasteurella*, *Edwardsiella*, *Chryseobacterium Pseudomonas*, *Corynebacterium*) (Hess et al., 2015). Though the microbiome was not characterized, a study with Atlantic salmon found that gill mucus cell count increases with salinity and fluctuations in ion regulation — additional research is needed to determine whether gill microbiomes play an active role in ion regulation (Roberts and Powell, 2003).

In summary, there is an absence of studies quantifying fish gill microbiomes, and comparative studies systematically characterizing the coexisting microbiomes in gills and surrounding aquatic environments are lacking. Generally, gill microbiomes tend to have a different composition and lower richness and diversity compared to skin and water microbiomes (Wang et al., 2010; Lowrey et al., 2015; Legrand et al., 2018; Pratte et al., 2018); however, gill environments seem to be uniquely enriched in nitrogen-fixing bacteria from the excretion of ammonia through the gill epithelia. Though it requires more invasive sampling methods, the gill microbial environment is affected by disease status and may provide valuable knowledge of overall animal health.

### Skin Microbiome

Fish epithelial surfaces are coated in mucus that provides a barrier between the host and aquatic environment. Skin mucus is an important component of fish immune systems and contains various immunoglobulins, mucins, antimicrobial peptides, and other mucosal biomolecules that protect the fish from pathogens (Gomez et al., 2013). Skin mucus hosts a diverse community of commensal microorganisms, namely bacteria but also archaea and fungi, believed to play a role against infection. For example, probiotic treatment with skin bacteria isolated from brook char (*Salvelinus fontinalis*) reduced pathogenic infection by up to 84% (Boutin et al., 2012). Microbial isolates from skin of rainbow trout (*Oncorhynchus mykiss*) inhibited the growth of skin fungal pathogens (Lowrey et al., 2015). Fish with internal diseases, like enteritis, display differences in diversity and enrichment of skin and gill microbial phyla compared to healthy fish (Legrand et al., 2018). The dynamic role of skin microbiomes in disease in fish make them a useful indicator of individual health, and the less invasive nature of skin sampling methods (fin clips, mucosal swabs) makes them beneficial for studying wild and/or protected species.

Fish skin microbiomes are distinct from those of the surrounding water in both composition and diversity, a pattern also observed in marine mammals, as discussed in a later section. A study with cultured gibel carp and bluntnose black bream found that bacterial communities in rearing pond water were more diverse than those in the skin of carp, but less diverse than in the skin of bream. Additionally, the diversity of specific groups, such as Actinobacteria and fungi, was higher in skin compared to water (Wang et al., 2010). A study assessing skin microbiomes in 44 species of reef fish saw high compositional dissimilarity between seawater and skin microbiomes. For example, planktonic communities were observed to be enriched in Cyanobacteria and Archaea, while bacteria on skin were enriched in Proteobacteria and Firmicutes. Skin microbiomes from the studied species of reef fish were more diverse than seawater, hosting twice as many classes and phyla, and only 10% of skin OTUs were also detected in seawater (Chiarello et al., 2018). In another study, approximately 7% of skin OTUs in European seabass (*Dicentrarchus labrax*) and gilthead seabream (*Sparus aurata*) were found in water samples, with water communities having a higher taxonomic richness due to the presence of rare OTUs (Chiarello et al., 2015). Free-living bacteria present in water communities do not colonize surfaces, which may account for some of the differences between microbiomes of tissues and the surrounding water (Dang and Lovell, 2016), while others could be related to species-specific coevolution and host-specific variation (Gomez et al., 2013; Uren Webster et al., 2018). Despite these differences, the diversity of water microbiomes affects fish skin microbiomes, as seen in comparisons between skin communities of wild-caught and captive-raised fish of the same species (Uren Webster et al., 2018) as well as comparisons between freshwater and marine fish.

Skin bacteria vary significantly among species, even those occupying the same environments. A study characterizing skin microbiomes of fish from the Gulf of Mexico reported distinct clustering of microbial profiles among species. For example, though the skin of the fish species examined was dominated by Proteobacteria, species-specific variation occurred among the Proteobacteria classes Alpha, Beta, and Gammaproteobacteria. Abundance of less frequent phyla like Bacteroidetes, Firmicutes, Actinobacteria, and Cyanobacteria varied considerably among species (Larsen et al., 2013). In a study with European seabass (*Dicentrarchus labrax*) and gilthead seabream, skin microbiome composition differed by 70% between the species (Chiarello et al., 2015). The skin microbiomes of two freshwater Amazon River fish (flag cichlids, *Mesonauta festivus*, and black piranhas, *Serrasalmus rhombeus*) were different from each other, with fish species accounting for 26% of the variation (Sylvain et al., 2019). Chiarello et al. (2018) found that among 16 species of fish from two reefs, fish species was significantly associated with skin microbial dissimilarity. Notably, in the same study, diet was the only ecological trait affecting skin microbiome structure, highlighting the important interaction between host diet and its microbiota.

Interrelated environmental conditions such as water quality and chemistry, geographic location, and season can also influence the skin microbiome in fish. In Atlantic cod (*Gadus morhua*) sampled in spring/fall 2002 and spring 2003, while skin seasonal microbial diversity and OTU composition was stable in 2 of 3 sampling sites, North Sea fish saw an increase in diversity from 2002 to 2003 and the three seasons shared only 8 out of 41 OTUs in common. In the same study, skin microbiome composition was also influenced by geographic location. Though Gammaproteobacteria and Bacteroidetes dominated skin microbiomes of cod from all three locations, the mean abundance of various genera, like *Psychrobacter* and *Photobacterium*, was changed across sampling sites. The presence of less-abundant phylotypes was also site-specific (Wilson et al., 2008). Phylogenetic structure of skin microbiomes was dissimilar among fish of the same species but from different reefs (Chiarello et al., 2018). European catfish (*Silurus glanis*) skin microbiomes assessed in France differed by sampling site in both diversity and community structure (Chiarello et al., 2019).

As aquatic bacteria have differing salinity tolerances, salinity may also affect skin microbiomes. Wild eels (*Anguilla anguilla*) from freshwater and estuarine environments displayed considerable variation in skin microbial composition, with higher salinities (3–10 g/L) enriching genus *Vibrio* within Gammaproteobacteria and lower salinities (≤ 1 g/L) enriching genus *Sphingobium* within Alphaproteobacteria and increasing OTU diversity in general (Carda-Diéguez et al., 2017). The shift in *Vibrio* bacteria associated with increasing salinities was also observed in Mexican mollies (*Poecilia sphenops*), where low salinities were associated with enrichment of *Aeromonas* within Gammaproteobacteria. The authors report approximately 44% of homogenate OTUs were correlated with changing salinity (Schmidt et al., 2015). Many *Vibrio* species are pathogenic to fish; thus, shifts in skin *Vibrio* abundance due to rising regional seawater salinity related to anthropogenic surface warming (Durack et al., 2012) could induce pathogenesis in certain fish species.

In addition to salinity, dissolved oxygen and nutrients also affect fish skin microbiomes. Recently published research with freshwater fish in the northern Jordan River found alterations in skin microbial phyla associated with anthropogenically interrupted aquatic systems. Specifically, the authors noted a shift from Proteobacteria to Bacteroidetes in the skin of fish from nutrient-polluted sites (Krotman et al., 2020). In the same study, dissolved oxygen weakly correlated with differences in microbial diversity among fish of different sites. In the Amazon River, water color (white, eutrophic vs. black, oligtrophic waters) accounted for almost 40% of skin microbiome variance between two Amazon fish species, with oligotrophic waters decreasing abundance of Alphaproteobacteria and increasing abundance of class *Mollicutes* within Firmicutes (Sylvain et al., 2019). Though more research is necessary regarding the effect of eutrophication aquatic microbial communities, these studies suggest that human organic and nutrient pollution in surface waters could lead to enrichment of certain phyla in fish skin microbiomes.

In summary, skin microbiomes play an important role in immunity in fish and there are differences in composition between different species and environments. The skin of marine fish is often colonized by Proteobacteria, especially Alphaproteobacteria and Gammaproteobacteria (e.g., *Psychrobacter and Photobacterium*). Other phyla common to fish skin microbiomes include Firmicutes, Actinobacteria, and Bacteroidetes, such as *Flavobacterium* (Wilson et al., 2008; Larsen et al., 2013; Chiarello et al., 2015, 2018; Sylvain et al., 2019). Identifying core microbial taxa colonizing fish skin is an important step toward establishing microbiomes as population health indicators. Due to the less-invasive nature of skin microbiome sampling compared to that of guts and gills, exploring the role of the skin microbiome as a health indicator in fish could aid in method refinement and reduction of animal harm.

### Gut Microbiome

Like mammals, fish have complex intestinal microbiomes that aid in nutrient absorption, immune system function, intestinal development, energy homeostasis, and xenobiotic metabolism. The acidic environment of the intestines favors certain yeasts and gram-negative bacteria. Gut microbial composition is highly influenced by species and diet as well as environment (Nayak, 2010; Sullam et al., 2012; Vasemägi et al., 2017; Adamovsky et al., 2018).

The gut microbiome is important for immune function. Some gut inhabitants of fish display inhibitory activity against pathogens. *Carnobacterium* spp. isolated from the Atlantic salmon intestine had *in vivo* antimicrobial effects on seven bacterial pathogens, and fish fed comprising *Carnobacterium*-enriched diet for at least 14 days had increased survival compared to controls after pathogen challenge (Robertson et al., 2000). Approximately, 28% of 400 bacteria isolated from the intestine of farmed turbot (*Scophthalmus maximus*) inhibited the marine pathogen *Vibrio anguillarum* while 60% of the intestinal isolates also inhibited other fish pathogens (Westerdahl et al., 1991). The gut microbiome may be more sensitive to changes in disease status and susceptibility than other microbiomes. Rainbow trout genetically resistant to the salmonid pathogen *Flavobacterium psychrophillum* had significant differences in gut microbial diversity, composition, and richness compared to susceptible fish, but the same differences were not observed in gill microbiomes (Brown et al., 2018). Gut microbial diversity is also correlated with *Tetracapsuloides bryosalmonae* infection, a trout endoparasite, in wild brown trout (*Salmo trutta*) (Vasemägi et al., 2017), highlighting the sensitivity of gut microbiomes to immune stress.

The fish gut microbiome is greatly influenced by feed types and metabolism of nutrients. Numerous bacteria isolated from the gut of fish, especially anaerobic *Bacillus* and *Aeromonas*, produce metabolic enzymes such as lipases, proteases, and amylases (Ray et al., 2012). Indeed, probiotic modulation of the gut microbiome of zebrafish larvae with *Lactobacillus rhamnosus* increased the ratio of Firmicutes to Actinobacteria, altering genes and phenotypes related to lipid metabolism and processing (Falcinelli et al., 2015). Fast-growing transgenic carp (*Cyprinus carpio*) had a different gut microbial composition, increased carbohydrate metabolism, and decreased lipid metabolism compared to wild-type fish. Specifically, transgenic carp had a lower gut ratio of Bacteroidetes to Firmicutes (Li et al., 2013). Zebrafish fed a diet enriched with phosphate-containing nucleotides exhibited increased growth and a lower metabolic rate than controls, and when the microbiome of nucleotide-fed zebrafish was transplanted into larvae, those fish displayed a similarly low metabolic rate compared to germ-free controls (Guo et al., 2017). The metagenome of gut microbiomes in grass carp (*Ctenopharyngodon idella*) reflected diet-related differences in nutrient metabolism (Ni et al., 2014), further highlighting the connection between the gut microbiome and nutrient status in fish. The composition of gut microbiomes in fish varies with diet and species, though diet seems to be a stronger driver (Sullam et al., 2012; Riiser et al., 2020). In reef fish, gut microbiomes clustered based on diet, regardless of species. Carnivores displayed the most separation from herbivore and omnivore microbiomes while the latter two were more similar to each other (Pratte et al., 2018). Miyake et al. (2015) reported that diet influenced the composition of gut microbiomes in Red Sea surgeonfish, parrotfish, and rabbitfish, but that a core microbiome among diet classes could not be identified. There was significant inter-species variability in gut microbial communities at both the phylum and genus level. In herbivorous fish, gut microbiomes are usually dominated by Bacteroidetes and Firmicutes, specifically those within order Clostridia, such as *Epulopiscium* (Clements et al., 2007; Smriga et al., 2010; Sullam et al., 2012; Miyake et al., 2015). Carnivorous fish in both freshwater and marine environments have abundant intestinal Proteobacteria, often in orders Enterobacteriales and Vibrionales (Kim et al., 2007; Ward et al., 2009; Smriga et al., 2010; Sullam et al., 2012; Xing et al., 2013; Tarnecki et al., 2016). Of course, there are exceptions to these trends, as exemplified in fish with high levels of phylum Tenericutes, like the king mackerel (*Scomberomorus cavalla*) (Givens et al., 2015) and zebrafish (Roeselers et al., 2011), and the high abundance of gut Proteobacteria in sharks (Givens et al., 2015). The importance of animal diet in the compositional structure and function of the gut microbiome is further highlighted in the section “Marine Mammal-Associated Microbiomes, subsection Gut Microbiome” and in the section “Microbiomes as Indicators of Host and Ecosystem Health.”

Environmental conditions such as habitat salinity and temperature can affect gut microbial profiles in fish. Gut microbiomes of farmed Atlantic salmon responded to seasonal changes in water temperature —Vibrionaceae (phylum Proteobacteria) dominated at higher temperatures (14–18°C) and disappeared at colder temperatures (10–12°C), while the reverse was true for lactic-acid bacterial species (phylum Firmicutes) (Neuman et al., 2016). Neuman et al. (2016) noted a decrease in metabolic dynamics of gut microbiomes in Atlantic salmon correlated with higher temperatures and *Vibrio* abundance. The seasonal shift from Proteobacteria to Firmicutes in salmon has implications for the potential impact of climate change on the microbiomes of aquatic species, especially those with thermosensitive processes. Habitat salinity may also shape the gut microbiome. Gut microbiomes in fish from freshwater and marine environments were distinct from each other, yet similar in composition to those of non-fish inhabiting environments of the same salinity (Sullam et al., 2012).

Water nutrient and dissolved oxygen levels may also impact gut microbiomes in fish. Sylvain et al. (2019) found that the diversity and composition of the gut microbiome in two Amazon fish (flag cichlid and black piranha) were changed in eutrophic versus oligotrophic waters. Specifically, fish from eutrophic waters had a higher diversity and abundance of Gammaproteobacteria, Alphaproteobacteria, and Oxyphotobacteria. Importantly, high levels of dissolved nutrients promote the growth of toxin-releasing cyanobacteria, which may impact the gut microbiome in fish, a prospect discussed further in the section “Cyanobacteria.”

Taken together, unique microbial communities exist in fish tissues, each of which function in tissue-specific physiology. In the aquatic environment, abiotic and biotic factors modulate microbial diversity, composition, and function, resulting in a complex and dynamic relationship between the external environment and the individual. These complex relationships are not unique to fish and extend to other aquatic vertebrates such as marine mammals. Marine mammals presented with significant challenges in terms of sampling and opportunity compared to fish, but emerging research has revealed an array of microbial diversity in species such as dolphins and whales. Below, we present what is known about microbial communities in marine mammal tissues, highlighting challenges and the potential implications for conservation efforts in these species.

## Marine Mammal-Associated Microbiomes

**FIGURE 2:**
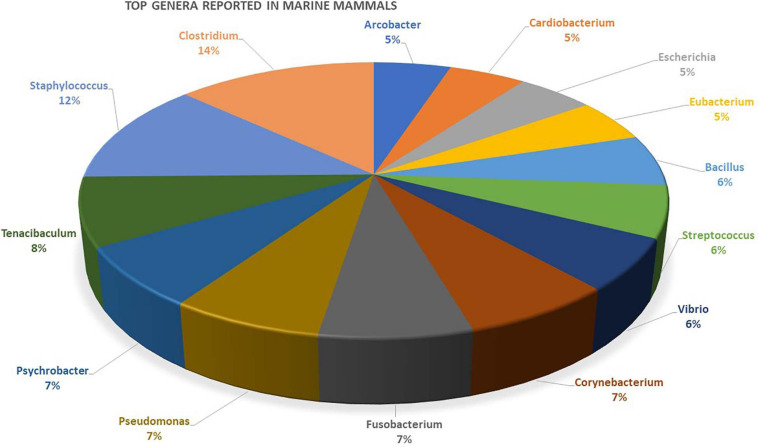
Genera most often reported in primary marine mammal microbiome studies from various tissue-specific niches. A list of the different genera reported in studies and details about the mammal’s host is provided as Supplementary Table 2.

In the conservation of marine mammals, analysis of the microbiome is a new and emerging non-invasive technique to assess health (Raverty et al., 2017; Hooper et al., 2018; Marón et al., 2019; Nelson et al., 2019). Only recently has microbiome research been incorporated into marine mammal conservation work on a significant scale (Hanning and Diaz-Sanchez, 2015; Pascoe et al., 2017; Comizzoli and Power, 2019). Studying the microbiome using non-invasive sampling is key to marine mammal research because many are elusive, protected, and/or too large for capture (Delport et al., 2016; Nelson et al., 2019). Currently, marine mammal microbiome research is working toward a foundation of data characterizing the microbial load of different marine mammal species with the goal of using the microbiome to set health biomarkers (Delport et al., 2016; Raverty et al., 2017; Bierlich et al., 2018; Hooper et al., 2018; Nelson et al., 2019; Soares-Castro et al., 2019; Suzuki et al., 2019a,b). In this section, we synthesize these findings from studies employing non-invasive collection techniques (Figure 2 and Supplementary Table 2). The discussion section will outline limitations and discuss the future trajectory of microbiome research in marine mammal conservation in relation to emerging environmental pressures.

### Respiratory Microbiome

Marine mammals have a unique respiratory anatomy as the nasopharynx is independent of the oral cavity; therefore, it may harbor rare microbes (Apprill et al., 2017). The blowhole of cetaceans is unique in that their respiratory system is directly exposed to both surface water and air microbiome when they take a breath, allowing for the microbiota composition to be influenced by external factors as well as host biological factors. This intersection of external and internal environment poses an interesting research opportunity. Bik et al. (2016) sampled both the blow hole mucosa (via swab) and chuff (exhalation caught in a filter) of bottlenose dolphins (*Tursiops truncatus*) and found higher microbial richness and diversity in the chuff. This suggests that the internal respiratory tissues host their own unique microbiome and blow is more than just aerosolized sea water (Bik et al., 2016; Apprill et al., 2017; Raverty et al., 2017; Vendl et al., 2019), which could have respiratory health and disease implications making this sampling method clinically relevant. However, researchers reported that the blow microbiome of four captive common bottlenose dolphins (*Tursiops truncatus)* was not distinct from that of the pool water (Nelson et al., 2019). It is currently unknown if this is due to captivity (versus wild mammals), water filtration practices, or species differences. Apprill et al. (2017) identified 25 core taxa (host-specific) in blow samples from humpback whales off the coast of Vancouver, Washington state, and Massachusetts including genera such as *Corynebacterium* (Actinobacteria phylum), *Tenacibaculum* (Bacteroidetes phylum), *Moraxella* (Proteobacteria phylum), and *Psychrobacter* (Proteobacteria phylum). Conversely, Vendl et al. (2019) found poor microbiota richness and a small core microbiome in humpback whales off the coast of Australia. The authors hypothesized that the lack of core microbiome may be related to the physiological state of the animals at the time of sampling. The animals sampled in Vendl et al. (2019) were 4 months into their migration which is a time of fasting; whereas, the animals sampled in the study by Apprill et al. (2017) were at their feeding grounds and early on into their migration. The difference between metabolic states may explain the difference in core microbiota found in humpback whales. Core microbiome of healthy animals are thought to serve beneficial roles to the animal hosts; therefore, identifying core taxa may allow researchers to quickly and non-invasively identify unhealthy individuals by assessing abnormalities of blow microbiota (Apprill et al., 2017; Vendl et al., 2019). Theoretically, the established core microbiome would be used as a reference to identify diseased animals, assuming the presence of a core microbiome is an indicator of good health.

### Oral Microbiome

Studying the microbiome of the oral cavity with swabs in captive and wild animals is an attractive option due to accessibility and non-invasive sampling methods. Researchers have been interested in determining whether the oral microbiome is distinct from the seawater. Bik et al. (2016) found that the seawater microbiome was largely different in composition from the oral microbiome of bottlenose dolphins (*Tursiops truncates*) and California sea lions (*Zalophus californianus*). They found that the fish and squid diet of dolphins and sea lions were also microbially distinct. The contrast in microbiome of seawater, diet, and mouth could indicate that the oral microbiota composition is host-specific. The oral microbiome was found to be very diverse with a low number of core taxa in common dolphins (*Delphinus delphis*), striped dolphins (*Stenella coeruleoalba)*, harbor porpoises (*Phocoena phocoena*) (Soares-Castro et al., 2019) and common bottlenose dolphins (*Tursiops truncates*) (Bik et al., 2016). Bik et al. (2016) found 25 phyla represented in the oral microbial community of wild (*n* = 10) and captive (*n* = 38) dolphins of the US Navy marine mammal program (MMP) collectively. When compared, the oral microbial compositions from the two dolphin populations (captive and wild) were significantly different potentially due to diet, location, and social behavior. Moreover, the oral microbial composition of California sea lions (*Zalophus californianus*) (*n* = 18) of the MMP was distinct from the dolphins of the same program. These findings suggest that microbiome composition is dependent on host physiology. On the other hand, the oral microbiome may be too variable to have an intra-species core microbiome as evidenced from a study on Odontoceti cetaceans (*n* = 48), which observed a small number of core OTUs and divergence between species (Soares-Castro et al., 2019). This difference in microbial communities within individuals was attributed primarily to habitat use, location, and developmental stage of the animals. Therefore, even though the oral microbiome seems to be influenced by host physiology and is distinct from the surrounding environment the oral microbiome may not be worth pursuing as a reliable a biomarker of the pinniped and cetacean health.

### Skin Microbiome

The skin microbiome is an important area of study in marine mammal research and perhaps the most accessible to sample. In recent years, the use of biopsy darts for sampling skin tissues from healthy cetaceans swimming swiftly in the marine habitats has advanced research understanding their skin microbiome diversity and health status with changing environmental conditions. In the past, researchers relied on stranded unhealthy animals to collect skin for microbiome analysis (Apprill et al., 2011, 2014; Chiarello et al., 2017; Bierlich et al., 2018; Russo et al., 2018; Hooper et al., 2019). There is a close mutual interaction that occurs between the skin epithelium and microbes that inhabit the water column. Similarly to fish, temperature, water chemistry, skin sloughing, season, diet, physiological state, geographic region, and horizontal and vertical host inheritance may all also affect the skin microbiome of marine mammals (Apprill et al., 2011, 2014; Chiarello et al., 2017; Bierlich et al., 2018; Russo et al., 2018; Hooper et al., 2019).

Studies have shown that even though the skin microbiome is constantly exposed to the external environment, it is genetically distinct from its external environment (Apprill et al., 2011, 2014; Chiarello et al., 2017; Hooper et al., 2019). This is thought to be due to individual animal epithelium, surface substrates, physiological state, and immune response (Apprill et al., 2011, 2014) similar to what has been described in fish (Gomez et al., 2013). Studies with wild humpback whales found that skin microbial composition was less diverse than taxa found in the surrounding water (Apprill et al., 2011, 2014). However, a study by Chiarello et al. (2017) on captive killer whales and dolphins reported that the skin microbiome was more diverse than the pool water. This may be due to the fact that the Mediterranean seawater that fills the pools is filtered, which may change or deplete the microbial load. The animals may also not be able to perform behaviors like jumping, swimming fast, and migrating to different water temperatures that would encourage skin sloughing and reduce microbial load (Chiarello et al., 2017). Skin sloughing is dead skin leaving the animal’s body due to natural skin turnover or a behavior, like jumping that forcibly removes dead skin due to impact with surface water. The different physiological states and properties of older and newer skin may affect microbial colonization (Apprill et al., 2014). Antibiotics often used to treat animals in captivity can alter the natural microbial composition (Chiarello et al., 2017). Human contact with captive animals is also likely to contribute to, and possibly increase, skin microbiome diversity.

Several studies have found evidence of a core microbiome from skin samples, and some are even conserved throughout regions within the same species (Apprill et al., 2014; Bierlich et al., 2018; Hooper et al., 2019). Hooper et al. (2019) found a highly related overlap of microbial taxa between animals of separate ecotypes. Similarly, Apprill et al. (2011) identified a core microbiome in 8 healthy humpback whales. Bierlich et al. (2018) sampled 89 humpback whales in different regions along the Western Antarctic Peninsula and found several core taxa conserved across all samples, independent of regional and seasonal variations: *Psychrobacter*, *Tenacibaculum*, uncultured Moraxellaceae, *Flavobacterium*, Flavobacteriaceae, and Gracilibacteria. Foraging season may have a profound impact in core microbiome presence in marine mammals, as evidenced by the difference in core microbiota in humpback whale skin (Bierlich et al., 2018) and blow (Apprill et al., 2017) sampled at different foraging and fasting times. Interestingly, season was related to changes in microbial composition and late season samples were more diverse than early season (Bierlich et al., 2018). It should be kept in mind that some, if not all, microbes colonizing external skin are responsive to environmental changes, which can affect microbiome data collected. For example, *Psychrobacter* sp., extremophiles of low temperature environments, are reduced in relative abundance with seawater temperature changes between seasons (Hooper et al., 2019).

Elucidating the symbiotic relationship between host and microbiome is an essential step in fully understanding the health services useful in establishing biomarkers. Currently, the functions of microbial taxa inhabiting marine mammals are largely unknown. Flavobacteriaceae, a family which includes genera *Tenacibaculum* and *Polaribacter*, was identified as part of the core genera of whale skin microbiome and is thought to be primarily commensal with marine organisms (Bowman and Nichols, 2005). In general, this family of bacteria is important in mineralization of organic matter in the surrounding marine environment (Bowman and Nichols, 2005). This function may be an important connection between host skin health and environmental health through a response to water quality. Moraxellaceae microbes are often found in animal mucosal membranes (Juni and Bøvre, 2015). Based on evidence found in rainbow trout, it is possible that *Psychrobacter* sp. (Moraxellaceae family) also exhibits antifungal properties on the humpback whale skin (Lowrey et al., 2015). Some species of the cold-tolerant *Polaribacter* genus (Flavobacteriaceae family) are pathogenic in fish (Rosado et al., 2019), but others are known to have important antioxidant functions (Sun et al., 2020). These core taxa confer important potential benefits and risks for humpback whale skin that necessitates further investigation to determine the nature of risks imposed by changing environmental conditions and their interactions with the host.

Noteworthy is that there are core genera common in marine mammals from different environments. For example, Gammaproteobacteria genus *Psychrobacter* was highly prevalent in captive killer whales and bottlenose dolphins (Chiarello et al., 2017) and wild humpback whales (Apprill et al., 2014; Bierlich et al., 2018). *Psychrobacter* was also one of the top 20 most abundant genera found on skin biopsies from offshore bottlenose dolphins but was not reported in onshore bottlenose dolphins (Russo et al., 2018). The skin of marine fish is also frequently colonized by the *Psychrobacter* (Apprill et al., 2011, 2014; Chiarello et al., 2017; Bierlich et al., 2018), highlighting the importance of this class to epidermal health in aquatic animals. There are other genera frequently detected on the skin of marine mammals. For example, *Tenacibaculum* was highly prevalent in humpback whale skin biopsy samples (Apprill et al., 2014; Bierlich et al., 2018) and was found in 95% of individuals sampled (Apprill et al., 2011). The high prevalence of *Tenacibaculum spp.* across populations of the same marine mammal species is evidence of a conserved core microbiome, although this high prevalence may be cause for concern. Tenacibaculosis, caused by *Tenacibaculum maritimum*, results in severe external lesions and necrosis in many marine fish species (Apprill et al., 2011; Pérez-Pascual et al., 2017; Guardiola et al., 2019). However, the high prevalence of *Tenacibaculum* in healthy marine mammals suggests a symbiotic rather than pathogenic relationship, as in fish (Apprill et al., 2014), because other species of *Tenacibaculum* have been found to be bacteriolytic and may fend off pathogenic colonizers (Banning et al., 2010). In summary, identifying the diversity and functionality of core microbiota conserved within a species and across ecotypes would enable better evaluation of animal health status and potential impacts of hydro-climatic changes.

### Gut Microbiome

Marine mammal gut microbiome studies are primarily done by collecting fecal samples from living wild or captive animals. Many studies of protected marine mammals are restricted due to a lack of access to animals and small population sizes. To compensate for this, researchers often collect samples from many populations of the same species. This offers an opportunity to compare microbial composition between disparate populations. Three main techniques are used. (1) Fecal samples are collected from the environment and then sampled from the center non-contaminated portion (Delport et al., 2016; Suzuki et al., 2019b). (2) Rectal swabs are also used on live animals that are either captive or temporarily captured in the wild (Bik et al., 2016). (3) Stranded, often deceased, animals are sampled directly from intestines. There is reasonable concern that the gut microbiota may change postmortem. However, studies have shown no significant difference in microbial composition related to state of decomposition in samples from fresh to moderately decomposed animals (Erwin et al., 2017; Marón et al., 2019). Although species richness was not profoundly affected by advanced decomposition, there was an increase in *Erysipelothrix* and a decrease in *Cetobacterium* in stranded Southern right whales (Marón et al., 2019). Despite limitations, deceased stranded animals present a unique opportunity to capture the microbiome of different physical and chemical sections within the intestines (Sanders et al., 2015; Wan et al., 2018). Wan et al. (2018) determined that fecal samples collected from five East Asian finless porpoises (*Neophocaena asiaeorientalis sunameri*) represented a higher percentage of anaerobic bacteria in the hindgut and fecal samples which was different than the community in the forestomach and foregut. Thus, fecal samples, while essential to gut microbial research, may provide a partial view of the diversity of gut microbial communities.

Diet is a predominant factor that determines gut microbial composition. Fermentation of plant and animal derived carbohydrates has been shown to be an important function in the gut microbial communities of several marine mammal species (Sanders et al., 2015; Erwin et al., 2017; Pacheco-Sandoval et al., 2019; Suzuki et al., 2019b). Ruminococcaceae, which specializes in plant cellulose degradation (Ezer et al., 2008), has been identified in Florida manatees (*Trichechus manatus latirostris*) (Merson et al., 2014; Suzuki et al., 2019b), Australian sea lions (*Neophoca cinerea*) (Delport et al., 2016), and baleen whales (Sanders et al., 2015). The genera *Faecalibacterium* and *Oscillospira* of the Ruminococcaceae family are anaerobes that perform fermentative metabolism; these were found in Pygmy and dwarf sperm whales (Erwin et al., 2017). Similar to reports in fish, Erwin et al. (2017) and Pacheco-Sandoval et al. (2019) found a higher percentage of Firmicutes than Bacteroidetes and both suggest the difference is due to diet. Data from the Pacific harbor seal (*Phoca vitulina richardii*) shows a positive correlation between anchovy consumption and presence of Firmicutes (Pacheco-Sandoval et al., 2019). Pacheco-Sandoval et al. (2019) hypothesized this is due to the high lipid content in anchovies and the lipid metabolic capacity of Firmicutes microbes. Dietary chitin from prey species also influences the gut microbiome as seen in Pacific harbor seals and baleen whales (Sanders et al., 2015; Pacheco-Sandoval et al., 2019). Chitin, made of polysaccharides, is the fibrous exoskeleton of invertebrates (Pacheco-Sandoval et al., 2019). *Bacteroides* and *Clostridium* are both chitinolytic bacteria found in Pacific harbor seals. *Bacteroides* were also found in baleen whale gut microbiome (Sanders et al., 2015). These microbes are most likely present to break down the chitin into nutrients readily absorbed by the host. According to Sanders et al. (2015) carbohydrate fermentation is essential to the gut microbiome of baleen whales, evidenced by an abundance of carbohydrate active genes related to both animal and plant carbohydrates. In summary, gut microbiome diversity and composition could be a good indicator of diet and nutrition in marine mammals but more research is needed in natural populations to establish robust relationships between diet and host microbiota.

Geographical location may determine differences in gut microbiomes between marine mammal populations. Delport et al. (2016) compared the gut microbiomes of Australian sea lions of geographically distinct wild populations and three separate captive populations. Their data suggested that the differences in diversity observed between these wild populations may be due to several factors such as, proximity to humans and colonies of different species, pollutants, waste water runoff, foraging location, diet, colony member dynamics, and behavior. The difference in microbial community membership of wild and captive Australian sea lions was not statistically significant. It is surprising that wild populations and captive populations would have similar microbiomes considering their vastly different habitats, diets, social interactions, and behaviors. This may support the idea of the role of phylogenetic relationships between host and body microbiome. The concept that the host microbiome is heritable, through vertical transmission, coevolves, and even adapts with the host is an emerging and complex topic (Nelson et al., 2013; Hauffe and Barelli, 2019). This concept may explain why populations of the same species in different habitats and geographical areas may have mirroring microbiomes. However, additional research is needed to conclusively determine factors that could cause the captive and wild populations to share microbiomes.

Other studies have investigated within and between population variability in the microbiome in marine mammals. Data from a study on stranded southern right whale calves (Eubalaena australis) shows three genera were possibly site specific (Marón et al., 2019). Genera *Allobaculum* and *Sarcina* were more prevalent in samples from Gulfo San José while the genera *Oscillospira* was only found in samples from Gulfo Nuevo. Two Weddell seal populations geographically isolated by an ice shelf in Antarctica differed in gut microbial diversity and composition (Banks et al., 2014). Seals of White Island have lower microbial diversity than those of McMurdo Sound. This difference in gut microbiome reinforces the theory that these seal populations are not freely mixing. The difference in microbial community between the two populations could be due to diet, body size, and/or population size (Banks et al., 2014). The smaller population size does not offer as many opportunities for mixing and transferring microbes. However, in a recent study by Pacheco-Sandoval et al. (2019) the smallest population of pacific harbor seals had the most diverse gut microbiome. This could possibly be due to the colony’s proximity to human development and runoff waters. In contrast, the fecal microbial communities from common bottlenose dolphins captive at the US Navy Marine Mammal Program in California were compared to those of wild bottlenose dolphins in Sarasota Bay, FL and there was no significant difference (Bik et al., 2016). This is unexpected considering the difference in geographical location, diet, medical care, human exposure, and findings from similar fish studies. The gut microbial community of hatchery-raised juvenile Atlantic salmon (*Salmo salar*) was also reported to be less diverse and had different community structure and function from the wild fish (Uren Webster et al., 2018). Taken together, the primary conclusion is that there can be notable differences between gut microbiomes of geographically separate populations due to habitat, social behavior, and diet.

To summarize, there are exciting applications for microbiota research in marine mammals from a conservation perspective. By comparing fish and marine mammals in terms of their tissue-specific microbiome, we have learned that the anatomy related to the respiratory tract and other tissues like skin can differ in microbiome composition, further evidence that microbial communities perform specific functions in different aquatic vertebrates. Moving forward, elucidating microbial diversity in species inhabiting the same aquatic microhabitat will be significant in understanding how environmental factors drive microbial communities; are these microbe communities more dependent upon an individual physiology and health (host-specific effects), evolutionary history of the species, or rather driven by environmental factors within local habitats (e.g., salinity and temperature). In the next section, we provide two examples of emerging stressors that can impact microbiomes of both fish and aquatic mammals.

## Emerging Stressors for Host-Associated Microbial Communities

Previous sections discuss the key role of the microbiome in host health and the factors that affect microbiome composition and diversity. Although there are many emerging contaminants that are priorities for scientific investigations and policy makers (e.g., pharmaceuticals, pesticides, cyanobacterial toxins, plastic additives and plastic waste), there is a scarce information regarding their effect on host associated microbiomes, specifically for aquatic organisms. Recent reviews investigating impact of pollutants on microbiome highlight that microbiome response is a key but underestimated element to better understand the toxicity of environmental contaminants on hosts (Adamovsky et al., 2018; Evariste et al., 2019; Duperron et al., 2020). Further, the direct effect of pollutants on microbial-driven nutrient cycling should not be ignored to characterize the consequences on ecosystem function. We selected two emerging stressors (microplastics and cyanobacteria) that are currently considered as top priority contaminates worldwide for aquatic environment due to human related activities. However, cyanobacteria and microplastics are frequently found in the environment and studied from different perspectives, and recent studies show their novel negative impact on host associated microbiomes.

### Cyanobacteria

Over the last few decades, cyanobacteria have become a significant environmental problem worldwide, due to their production of toxic compounds. Accelerated by climate change, cyanobacterial blooms are now an emergent global challenge. The US EPA reports that cyanobacteria are a major environmental problem in all 50 states, and toxic blooms can impact all aspects of environmental health (USEPA, 2020). Cyanobacteria produce numerous toxic secondary metabolites with hepatotoxic, neurotoxic, carcinogenic, endocrine, and immunomodulatory potency (Palikova et al., 2013, 2015; Adamovsky et al., 2015; Jarošová et al., 2015; Javu̇rek et al., 2015; Jonas et al., 2015; Moosova et al., 2018, 2019). Among these, microcystins (MCs), specifically microcystin-LR (MC-LR), are the most frequent and are present in high quantities in the environment (Bláha et al., 2010). Cyanobacteria produce compounds that affect other microbial organisms (e.g., induce oxidative stress) (Chen et al., 2015) that may interfere with bacterial quorum sensing, and exhibit allelopathic or antibacterial properties (Shah et al., 2017). Therefore, it is suggested that cyanobacteria may affect microbial communities in the environment but also host-associated microbiomes. Although studies with mammals show that MC-LR contributes to gut dysfunction by generation of reactive oxygen species (Gehringer et al., 2004), cell erosion, deficient intestinal absorption of nutrients and modulation of the immune system (e.g., increase of pro-inflammatory cytokines) (Adamovsky et al., 2011, 2015; Christen et al., 2013; Moosova et al., 2018, 2019), the role of the intestinal microbiome in cyanobacterial toxicity remains poorly investigated. Similarly, there are few studies exploring the effect of cyanobacteria on fish-associated microbiota (Lin et al., 2015; Duperron et al., 2019).

Recent pilot studies with MC-LR in rodents showed that MC-LR can significantly alter the mammals intestinal microbiome (Chen et al., 2015; Lin et al., 2015; Sarkar et al., 2020). Similarities among mammals in gut microbial structure indicate that marine mammal microbiomes may also be a target for cyanobacterial toxins. Chen et al. showed that the effect of intragastric administration of MC-LR on the microbiome significantly differs in different parts of the mice intestine. They observed significantly increased species richness in the cecum and colon, and significantly increased microbial diversity in the cecum after MC-LR exposure, but the microbiome in the jejunoileum remained unaffected (Chen et al., 2015). The effect on a specific part of the intestinal microbiome has potential functional consequences, as there is an extensive difference in the microbiome along the intestine (Crespo-Piazuelo et al., 2018). Additionally, other study with mammals showed microcystin-induced shift of the functional content of the microbiome (Lin et al., 2015), specifically changes in carbon degradation including chitin, starch, and limonene metabolism, and these enriched processes were mainly derived from fungal and bacterial pathogens. Similarly, other studies indicate that microcystin caused microbial dysbiosis similar to microbial shifts in diabetic mice (Chen et al., 2015) and in non-alcoholic fatty liver disease (NAFLD)-associated inflammatory bowel disease (Sarkar et al., 2020) including inflammatory pathology in the intestine, increased oxidative tyrosyl radicals, alternation of tight junctions and gut leaching, worsening manifestations of NAFLD (Sarkar et al., 2020). The ability of cyanobacteria to alter cell-cell junctions was also proven in *in vitro* studies (Nováková et al., 2011, 2013).

Though there is evidence that MC affects the intestinal microbiome in mammals, studies with fish revealed only minor changes of fish intestinal microbiomes caused by MC-LR exposure, indicating that the fish core microbiome is resistant to microcystins (Duperron et al., 2019; Li et al., 2019). In contrast, an exposure to a complex cyanobacterial extract of *Microcystis aeruginosa* had a significant influence on the fish gut microbiome, with a significant increase of pathogen-related bacteria (genera *Nocardia* and *Mycobacterium*) reported as abundant in animals with inflammatory bowel disease (Duperron et al., 2019). The cyanobacterial extract also increased *Saprospirales* and *Sphingomonadales*, which were found to use MC as a nutrient source and were isolated from environmental samples of surface waters (Ishii et al., 2004). Similarly, Li et al. (2019) identified the increase of the microcystin-degrading genus *Rhodococcus* in zebrafish exposed to MC-LR, implicating that the weak effect of pure MCs on fish microbiota is due to the ability of fish microbiome to degrade MCs (Ishii et al., 2004; Li et al., 2019). These adaptations may explain the different susceptibility of aquatic organisms to toxic cyanobacterial blooms. This was confirmed in a recent study with a crustacean (*Daphnia magna*) showing a strong difference in gut microbiota composition between MC tolerant and susceptible types, highlighting that microbiota is a significant driver of adaptation and acclimatization to cyanobacterial toxic blooms in zooplankton and potentially in other organisms (Macke et al., 2017).

In conclusion, cyanobacterial biomass is a mixture of bioactive compounds and toxins but current microbiome-related research is solely focused on one microcystin congener, MC-LR. More research is needed to investigate other cyanobacterial substances (e.g., lipopolysaccharides) with microbiome modulatory potency (Annadotter et al., 2005; Blahova et al., 2013; Salguero et al., 2019). MC-LR causes dysbiosis in mammals and fish have functional consequences, as an affected microbiome may alter feed efficiency, metabolism, immunity, pathogen susceptibility, or protective functions of the gut. More studies are needed to evaluate the impact of cyanobacteria-related microbial shifts on health and explore the characteristics of microbiomes resistant to cyanobacterial toxins. These interesting questions add another layer of complexity for investigations into microbiota of aquatic organisms. From the human perspective, there is a need to investigate the efficiency of current water treatment technologies at removal of cyanobacterial substances associated with gastrointestinal diseases, as studies indicate that specific water treatment plants are ineffective at removing toxins from cyanobacteria-polluted waters (Sovadinova et al., 2017).

### Microplastics

Plastic pollution in freshwater and marine environments has gained considerable attention over the past several years. Global plastic waste production is predicted to triple to nearly 270 million tons from 2015 to 2060 (Lebreton and Andrady, 2019). In the environment, plastics continuously break down into small fragments, called microplastics (MPs), usually defined as particles below 5 mm (Hartmann et al., 2019). MPs are found in almost all aquatic and terrestrial environments, where they become a part of the food chain. MPs are not only ubiquitous in the environment but have also been detected in human foods and drinking water (Zhang et al., 2020). MPs have a broad spectrum of chemical compositions, including the presence of additives, sizes, shapes, biofilm compositions, and conditions (pristine versus weathered) that strongly influence their environmental fate and potential toxicity to microbial communities.

Microplastics are ingested by a variety of aquatic organisms, from worms to whales (Browne et al., 2008; Lusher et al., 2013; Wright et al., 2013; Watts et al., 2014; Liu et al., 2019). Although MPs have been found in over 690 marine species (White et al., 2018), the majority of toxicological studies focus on fish. The increasing number of fish studies indicate that MPs not only cause physical damage, but can also affect reproduction, immune systems, metabolism, growth rate, block digestive tracts, induce oxidative stress, and cause dysbiosis (Caruso et al., 2018; Fackelmann and Sommer, 2019; Jin et al., 2019; Qiao et al., 2019; Wan et al., 2019; Jacob et al., 2020). Dysbiosis may affect health through alterations of immunologic activity, neurobehavioral development, gut performance, and development of chronic diseases. Though the intestinal microbiome has a key role in health and disease, to date only a few studies address the effect of MPs on host microbiomes. Recent research indicates that MPs, specifically pristine polystyrene (PS), polyvinyl chloride (PVC), and polyethylene (PE), have a potential to deregulate intestinal microbiomes in worms (Zhu et al., 2018), crustaceans (Liu et al., 2019), snails (Chae and An, 2020; Horton et al., 2020), mammals (Lu et al., 2018; Jin et al., 2019; Li et al., 2020), and fish (Caruso et al., 2018; Jin et al., 2019; Qiao et al., 2019; Wan et al., 2019). Investigations into MP-related microbiome alterations have only just begun and a few studies have explored the effect of MPs on fish intestinal microbiomes in controlled captive conditions (Qiao et al., 2019). MPs are also covered by biofilms and aged by abiotic and biotic processes. The low number of studies do not allow for systematic evaluation of the effect of PS microparticles on fish, but interestingly, PS microparticles induced similar microbial shifts among the studies. Exposure to PS microparticles (7–21 days) significantly decreased the relative abundance of *Proteobacteria* (Jin et al., 2018; Qiao et al., 2019; Wan et al., 2019) in zebrafish. Further, acute exposure (up to 2 weeks) to PS microparticles increased phylum *Firmicutes* and decreased phyla *Bacteroidetes* and *Proteobacteria* (Jin et al., 2018; Wan et al., 2019) in zebrafish. In addition, PS microparticles caused alterations in gut histology, induced oxidative stress and inflammatory response in the gut, and influenced energy and glycolipid metabolism (Qiao et al., 2019; Wan et al., 2019; Jacob et al., 2020).

Compared to controlled studies with pristine MPs, the situation in the real environment is more complex. Environmentally relevant MPs are always a mixture of different types of plastic materials with a broad spectrum of leaching additives (e.g., plastic softeners and UV stabilizers) that may contribute to adverse effects on microbiomes (Adamovsky et al., 2020). The environment and its biota are exposed to complex mixtures of micro/nano-plastics, their degraded products, adsorbed contaminants, plastic-associated chemicals, and plastic-specific bacteria. Bacteria growing on plastics appear to be polymer-specific, thus controlled experiments using one type of polymer, instead of a mixture, will promote colonization with a certain bacterial community structure including specific pathogens (Frère et al., 2018). Microbiome dysbiosis might be caused not only by MPs themselves, as shown in controlled studies, but the ingestion of potentially pathogenic bacteria and/or chemicals leaching from or adhering to plastics. Further, MPs were shown as a hotspot for plasmid-mediated gene transfer in bacteria. The authors hypothesize that pollution by microplastics in aquatic ecosystems favors higher transfer frequencies of plasmids carrying antibiotic resistance genes (Arias-Andres et al., 2018).

Though several pilot studies indicate that pristine MPs can affect host microbial communities, the research investigating complex, environmentally relevant MPs (i.e., aged and/or befouled MPs, environmentally relevant concentrations) and their ability to interfere with the host microbiome is still in its infancy. Importantly, MPs are ingested by many aquatic organisms, but the depuration kinetics is known only for a low number of specific plastic types and sizes, and speed of depuration strongly influences the potential of MPs to interfere with host microbiomes.

## Microbiomes as Indicators of Host and Ecosystem Health

As highlighted in previous sections, microbiomes are tightly linked to the functioning and health of ecosystems (e.g., animal health, nutrient recycling, water quality). A decade of microbiome research has revealed that these complex microbial communities respond rapidly to environmental disturbances in the form of biotic or abiotic pressures, sometimes in a matter of hours or days (Païssé et al., 2010; Landesman and Dighton, 2011). This sensitivity of microbiomes to environmental disturbances opens avenues for use of microbial communities as standard ecological indicators for biomonitoring of ecosystem or host health. In this section, we highlight the following four (I-IV) motivations that encourage the development of microbiome-based indicators in aquatic ecosystems.

(I) Microbiomes are ubiquitous. Microbes inhabit all types of ecosystems, even under extreme temperatures, salinities, or pollution. This is not the case for traditional ecological indicators, like macroinvertebrate communities (e.g., Ephemeroptera-Plecoptera-Trichoptera), widely used to monitor water quality but do not inhabit highly degraded ecosystems. Using microbiomes as bioassessment tools is potentially applicable to all types of ecosystems, including extreme environments (e.g., hypersaline, low pH environments) or highly degraded sites (e.g., mining sites, pollution spills), where traditional biomonitoring tools may not be effective.

(II) Microbiota analysis can be high-throughput at a low cost. Combining environmental DNA (eDNA) with sequencing in biodiversity monitoring is now used to characterize natural communities across the tree of life (insects, plant, fish, amphibians) (Taberlet et al., 2012; Bohmann et al., 2014; Rees et al., 2014). The low costs and sensitivity of eDNA-based surveys compared to morpho-taxonomic identifications (Cordier et al., 2019) is highly appealing. Even at a single site, traditional biodiversity sampling is time consuming, requires taxonomic expertise, and can harm organisms, while eDNA collection is much less invasive and can overcome any conservation or ethical considerations. Microbiome-based monitoring is accessible through the collection of eDNA and high-throughput sequencing using specific microbial primers (e.g., metabarcoding of 16S rRNA, ITS region or 18S rRNA gene). This approach presents the opportunity to monitor hundreds of sites/hosts at multiple time points in a single sequencing run, often not logistically or financially realistic with traditional biomonitoring methods.

(III) Microbiota analysis is versatile and historically informative. When individuals are exposed to new environmental conditions, the composition and structure of the microbiome can respond quickly, with high turnover particularly among sub-dominant or rare taxa (Shade et al., 2014). Recent studies suggest that the microbiome of an ecosystem or host can be divided into different components: a core microbiome, where microbial taxa are shared across multiple sites and environmental conditions (low number of taxa and highly stable), and the variable microbiome (also called accessory or flexible microbiome), which differs strongly with biotic and abiotic fluctuations of the environment (high number of taxa and low stability; Vandenkoornhuyse et al., 2015; Hernandez-Agreda et al., 2016; Simonin et al., 2020). This variable microbiome represents a vast pool of potential microbial indicators, either “generic” (sensitive to multiple stressors) or “specific” (respond to a specific pollutant or change). In parallel, analysis of the composition and stability of core microbiomes could provide indications of deep microbiome dysbiosis/reorganization within hosts (Carding et al., 2015; Cheng et al., 2020) or environments reflective of long-term stress to the ecosystem. Currently, most research focuses on the characterization of core microbiomes across many hosts and environments (Risely, 2020), but the development of effective microbiome-based indicators will depend on better characterization and understanding of the “variable” microbiome.

The use of microbial taxa as indicators across multiple systems is promising, as consistent microbial responses to the same disturbance (e.g., salinity, drought, elevated CO_2_) have been found to be phylogenetically conserved (Martiny et al., 2015; Isobe et al., 2020). For example, Isobe et al. (2020) demonstrated that phylogenetic information can be used to predict the response of bacterial communities to global stressors (e.g., elevated temperature, drought) and hence identify reliable bacterial indicators of these disturbances. In parallel, different initiatives aim to synthesize ecological knowledge about the habitat distribution and stress tolerance of microbial taxa at a global scale (e.g., Earth Microbiome Project, Microbiome Stress Project, Thompson et al., 2017; Rocca et al., 2019). The integration of this knowledge will enable the establishment of lists of microbial indicator taxa by disturbance type and biome, paving the way for large-scale use of microbiomes as bioassessment tools for natural and anthropogenic aquatic ecosystems.

(IV) Computation tools to identify microbial indicators and create microbiome-based biotic indexes are already available. Microbiome datasets can be complex to analyze and interpret due to the large diversity of these communities, which may be perceived as a constraint for development of mainstream microbiome-based monitoring. However, significant progress in bioinformatic pipelines to analyze metabarcoding and whole genome sequencing have made microbiome analysis more readily accessible (e.g., Qiime2, Anvi’o, Eren et al., 2015; Bolyen et al., 2019). Moreover, many statistical tools to identify responsive or indicator microbial taxa are available and have been adapted to microbiome data. Depending on the sampling design of the survey, different statistical methods for differential abundance testing or indicator taxa analysis (e.g., IndVal) are available. For instance, the Threshold Indicator Taxa Analysis (TITAN2, Baker and King, 2010) can identify bacterial indicator taxa (tolerant and sensitive taxa) to phosphorus pollution (LeBrun et al., 2018) or urbanization (Simonin et al., 2019) in rivers. TITAN2 can be used to identify microbiome-level sensitivity thresholds (e.g., 55 μg/L of total phosphorus or 12% urban development). These values are extremely important for monitoring purposes and informing protective environmental quality criteria.

With these promising findings, microbiome-based biotic indexes are currently being developed for application in routine biomonitoring to assess the ecological quality status of aquatic environments (Cordier et al., 2019). These biotic indexes classify environments based on species richness, composition, abundance, and/or functions of microbiomes in comparison to reference conditions (e.g., unpolluted environment, healthy host). Aylagas et al. (2017) developed a bacterial community-based index for assessing ecological status of estuarine and coastal environments based on 16S rRNA gene metabarcoding. Keeley et al. (2018) developed a multi-trophic metabarcoding biotic index based on three taxonomic groups (Foraminifera, bacteria, and eukaryotes) for the biomonitoring of benthic enrichments in sea-based fish farms. Following the example of the successful use of macroinvertebrates, fish, and diatom communities in bioassessment, biotic indices based on microbiome data will provide a sensitive and integrative tool for rapid environmental assessment, and help protect key ecosystem and host services delivered by microorganisms (Lau et al., 2015).

## Future Directions

Core microbiomes of healthy animals are beneficial to the host; therefore, identifying core (consistent and dominant) taxa as biomarkers will allow researchers to quickly and non-invasively identify unhealthy individuals by assessing abnormalities in niche microbiomes (Bowman and Nichols, 2005; Rosado et al., 2019). However, variability is expected. In particular, animal-associated microbiomes are strongly influenced by natural fluctuations in water quality (e.g., season, temperature, salinity) but also by sex, captive-state, life-stage, trophic level, diet, and phylogeny (Egerton et al., 2018). As reviewed, certain body locations (e.g., marine mammal oral) have high diversity and low core microbiome when compared to others (e.g., marine mammal skin and blow, fish skin and gills). These tissues with highly variable microbiomes and those that do not show conservation between populations may not be useful for the development of reliable biomarkers of ecosystem health. However, large fluctuations in composition of the microbiome that cannot be explained by natural variability may serve as an indicator of exposure to environmental stressors in aquatic vertebrates.

To describe microbiomes for a larger spectrum of organisms and develop an integrative view of the microbial diversity of a given ecosystem, additional studies with higher replication are needed to quantify intra- and inter-species microbiome diversity. Due to a high complexity of samples, there is a need for sampling methods which preserve the information about the genome and the biological system at the time of sampling. Sampling and storage methods significantly affect microbial composition, and can subsequently affect the interpretation of data. For example, gut microbial communities in fish differ by tissue location and type. Fecal microbiomes, often reported as a proxy for intestinal microbiomes, have a different microbial diversity, richness, and composition than microbiomes of intestinal mucus (Kim et al., 2007). Additionally, mucus microbiomes from different sections of the gut can also different significantly from each other (Xing et al., 2013). The same issues with sampling exist among studies characterizing fish skin microbiota, which can vary with body sampling location and method (Chiarello et al., 2015). Tissue sampling site may also affect the results of studies investigating gill microbiomes, i.e., sampling of gill filaments, rakers, or only the gill mucus. Additionally, a 2020 study attempted to reduce “background” water contamination of skin microbiota during catch-and-release sampling and found a significant effect on results (Krotman et al., 2020). As tissue and water microbiomes differ considerably, water contamination could confound results, and future studies should work to address this variable. Though there exists evidence of a “core” gut microbiome in certain fish species (Roeselers et al., 2011; Jones et al., 2018), intraspecific microbial variation can make core microbiomes difficult to elucidate, especially across environments and ecological niches (Riiser et al., 2020). Studies should keep differences between tissue and sampling locations in mind and ensure an appropriate number of replicates when making comparisons and attempting to establish core microbiomes.

Shifts in microbiome composition can promote or mitigate disease states in hosts. Scientists are now investigating the possibility of manipulating these microbial communities to improve host health and, in case of fish, their nutritional values. Potential fish probiotics include microalgae, yeasts, and both gram-positive and gram-negative bacteria (Akhter et al., 2015). Our review points to the fact that the literature on aquatic animal tissue microbiomes is still limited; nevertheless, a majority of research assessing the structure and function of fish and marine mammal microbiota has been conducted in the gastrointestinal system. Gut microbiomes play a significant role in animal diet and can provide information regarding both nutritional status and environmental niche. For example, herbivorous and omnivorous fish tend to have more diverse microbiomes than carnivorous fish (Xing et al., 2013), and gut microbiomes of herbivorous and carnivorous fish are similar in composition to herbivorous and carnivorous mammals, respectively. Gut microbiomes of omnivorous fish are more similar to planktonic and invertebrate microbial communities (Sullam et al., 2012), which could be due to the propensity of omnivorous fish to consume small invertebrates in addition to plants. Diet also drives microbiome composition in marine mammals, and carbohydrates fermented from ingested plant and animal material can shape microbial communities in several marine mammal species (Sanders et al., 2015; Erwin et al., 2017; Pacheco-Sandoval et al., 2019; Suzuki et al., 2019b). In addition to compositional evidence, current data on function supports the role of the intestinal microbiome on diet. Microbiome functionality can be quantified with metagenome prediction tools, such as PICRUSt (Langille et al., 2013), substrate utilization methods such as Biolog Microplates, and NMR-based characterization of intestinal metabolites. Mouchet et al. (2012) found that compositional differences in fish gut microbial communities driven by species, sampling site, and diet were not reflected in community substrate utilization and degradation potential. Fish with different microbiomes had similar functionalities. In bluegill (*Lepomis macrochirus*) with three different feeding habits, anaerobic substrate utilization differed between herbivores, planktivores, and benthivores (Uchii et al., 2006). Lastly, presence of intestinal and fecal metabolites such as specific amino acids, fatty acids, and phospholipids was affected by diet, not species, in coastal/estuarine fish from Japan (Asakura et al., 2014).

The recent rise in studies investigating host microbiomes have significantly expanded our knowledge on this topic, but there is still a gap in the understanding of fundamental principles of the bidirectional relationship between microbiota and host, as well the natural variability of microbiomes. Extensive research in wild populations as well as controlled laboratory studies will be necessary to reveal the disruptive potency of global stressors such as pollution and climate change, including ocean acidification, on host-associated microbial communities. Lastly, research assessing the connections between different aquatic animal microbiomes is sparse and will be important for characterizing tissue-specific microbiomes as indicators of organism health.

## Conclusion

In this review, we summarize current knowledge regarding the microbiome in aquatic environments and identify novel aspects and gaps in microbiome research. In addition to frequently studied intestinal microbiomes, we highlight the importance of other microbial communities and how they may coexist with fish and marine mammals. Although the scientific community has made strides in elucidating microbiome complexity and dynamics in oral, skin, and respiratory microbiomes, there remains a huge lack in systematic studies addressing the factors responsible for variation within and between freshwater/marine animal populations. We described several significant abiotic (e.g., temperature, water chemistry), biotic (surrounding microbiome, physiology, diet), and anthropogenic (pollution, contaminants) factors that shape various host-associated microbiomes with potential consequences for host and ecosystem health. We recommend standardizing sequencing methodologies and sampling techniques to eliminate variation in microbiome investigations. Additional studies and an appropriate number of replicates are needed to overcome inconsistencies and to draw stronger conclusions. Further, future studies should focus on the functional differences in microbiome, as observed compositional differences do not necessarily mean differences in function of the microbiome. Lastly, we identify several reasons demonstrating that microbiomes will become standard indicators of ecological status and health of aquatic individuals or communities. Microbiome-based monitoring in animal tissues is anticipated to be a sensitive and integrative tool for rapid environmental health assessments.

## Author Contributions

LS, EB-R, and AW reviewed the literature and drafted the manuscript with input from authors LB, JB, IL, CM, MS, and OA. All authors contributed to the article and approved the submitted version.

## Conflict of Interest

The authors declare that the research was conducted in the absence of any commercial or financial relationships that could be construed as a potential conflict of interest.
