# Supplementary material for: Microbiome Composition and Function in Aquatic Vertebrates: Small Organisms Making Big Impacts on Aquatic Animal Health
Source: Front Microbiol. 2021 Mar 11;12:567408. doi: 10.3389/fmicb.2021.567408 (PMC7995652; doi:10.3389/fmicb.2021.567408)
Supplement: Supplementary file 1 [file Table_1.DOCX]

**Supplemental Materials**

**Tab.S1: The list of the tissue specific microbial genera in studies with marine mammal.**

| **Microbial Genera** | **Captive/**  **Wild** | **Marine Mammal Species** | **Study Area** | **Sample microbiome** | **Publication** |
| --- | --- | --- | --- | --- | --- |
| **Acidovorax** |  |  |  |  |  |
|  | Wild | Common bottlenose dolphin (Tursiops truncatus) | Southern California Bight, USA | Skin microbiome | (Russo et al., 2018) |
| ***Acinetobacter*** |  |  |  |  |  |
|  | Captive | Common bottlenose dolphin (Tursiops truncatus) | National Aquarium, Maryland, USA | Blow microbiome | (Nelson et al., 2019) |
|  | Wild | Indo-Pacific bottlenose dolphins (Tursiops aduncus) | Shark Bay, Western Australia | Blow microbiome | (Nelson et al., 2019) |
|  | Wild | Common bottlenose dolphin (Tursiops truncatus) | Southern California Bight, USA | Skin microbiome | (Russo et al., 2018) |
| ***Actinobacillus*** |  |  |  |  |  |
|  | Wild | Common bottlenose dolphin (Tursiops truncatus) | Sarasota Bay, FL, USA | Gut microbiome | (Bik et al., 2016) |
|  | Captive | Common bottlenose dolphin (Tursiops truncatus) | US Navy Marine Mammal Program, California, USA | Gut microbiome | (Bik et al., 2016) |
|  | Wild | Indo-Pacific bottlenose dolphins (Tursiops aduncus) | Shark Bay, Western Australia | Blow microbiome | (Nelson et al., 2019) |
| ***Adlercreutzia*** |  |  |  |  |  |
|  | Wild | Pygmy (Kogia breviceps) and Dwarf (Kogia sima) sperm whales | North Carolina | Gut microbiome | (Erwin et al., 2017) |
|  | Wild | Florida manatee (Trichechus manatus latirostris) | Crystal River, FL | Gut microbiome | (Merson et al., 2014) |
| ***Aeromonas*** |  |  |  |  |  |
|  | Wild | Common bottlenose dolphin (Tursiops truncatus) | Southern California Bight, USA | Skin microbiome | (Russo et al., 2018) |
| ***Akkermansia*** |  |  |  |  |  |
|  | Captive | Antillean manatee (Trichechus manatus manatus) | Ocean Expo Park, Okinawa, Japan | Gut microbiome | (Suzuki et al. 2019) |
| ***Alteromonas*** |  |  |  |  |  |
|  | Wild | Southern Resident Killer Whale (Orcinus orca) | Salish Sea | Blow microbiome | (Raverty et al., 2017) |
| ***Anaerobiospirillum*** |  |  |  |  |  |
|  | Wild | Pacific harbor seal (Phoca vitulina richardii) | Baja California, Mexico | Gut microbiome | (Pacheco-Sandoval et al., 2019) |
| ***Anaerococcus*** |  |  |  |  |  |
|  | Captive | Common bottlenose dolphin (Tursiops truncatus) | National Aquarium, Maryland, USA | Blow microbiome | (Nelson et al., 2019) |
|  | Wild | Indo-Pacific bottlenose dolphins (Tursiops aduncus) | Shark Bay, Western Australia | Blow microbiome | (Nelson et al., 2019) |
| ***Anaerovorax*** |  |  |  |  |  |
|  | Wild | Common bottlenose dolphin (Tursiops truncatus) | Southern California Bight, USA | Skin microbiome | (Russo et al., 2018) |
| ***Anoxybacillus*** |  |  |  |  |  |
|  | Wild | Humpback whale (Megaptera novaeangliae) | Queensland, Australia | Blow microbiome | (Vendl et al., 2019) |
| ***Aquabacterium*** |  |  |  |  |  |
|  | Wild | Humpback whale (Megaptera novaeangliae) | Southeast Alaska, Hawaiian Islands, Gulf of Maine, USA and American Samoa | Skin microbiome | (Apprill et al., 2014) |
| ***Arcobacter*** |  |  |  |  |  |
|  | Wild | Humpback whale (Megaptera novaeangliae) | Massachusetts and Washington, USA Vancouver, CAs | Blow microbiome | (Apprill et al., 2017) |
|  | Captive | Common bottlenose dolphin (Tursiops truncatus) | National Aquarium, Maryland, USA | Blow microbiome | (Nelson et al., 2019) |
|  | Wild | Indo-Pacific bottlenose dolphins (Tursiops aduncus) | Shark Bay, Western Australia | Blow microbiome | (Nelson et al., 2019) |
|  | Wild | Common dolphins (Delphinus delphis), Striped dolphins  (Stenella coeruleoalba) and Harbour porpoises (Phocoena phocoena) | Northern and Western Atlantic Iberian coast | Oral microbiome | (Soares-Castro et al., 2019) |
| ***Arthrobacter*** |  |  |  |  |  |
|  | Wild | Southern Resident Killer Whale (Orcinus orca) | Salish Sea | Blow microbiome | (Raverty et al., 2017) |
| ***Arthromitus*** |  |  |  |  |  |
|  | Captive | Common bottlenose dolphin (Tursiops truncatus) and California sea lions (Zalophus californianus) | US Navy Marine Mammal Program, California, USA | Gut microbiome | (Bik et al., 2016) |
|  | Wild | Common bottlenose dolphin (Tursiops truncatus) | Sarasota Bay, FL, USA | Gut microbiome | (Bik et al., 2016) |
| ***Atopobium*** |  |  |  |  |  |
|  | Wild | Pacific harbor seal (Phoca vitulina richardii) | Baja California, Mexico | Gut microbiome | (Pacheco-Sandoval et al., 2019) |
| ***Bacillus*** |  |  |  |  |  |
|  | Wild | Southern Resident Killer Whale (Orcinus orca) | Salish Sea | Blow microbiome | (Raverty et al., 2017) |
|  | Wild | Humpback whale (Megaptera novaeangliae) | Queensland, Australia | Blow microbiome | (Vendl et al., 2019) |
|  | Wild | Southern Resident Killer Whale (Orcinus orca) | Salish Sea | Blow microbiome | (Raverty et al., 2017) |
|  | Wild | Southern Resident Killer Whale (Orcinus orca) | Salish Sea | Blow microbiome | (Raverty et al., 2017) |
|  | Wild | Southern Resident Killer Whale (Orcinus orca) | Salish Sea | Blow microbiome | (Raverty et al., 2017) |
| ***Bacteriodes*** |  |  |  |  |  |
|  | Wild | Pacific harbor seal (Phoca vitulina richardii) | Baja California, Mexico | Gut microbiome | (Pacheco-Sandoval et al., 2019) |
|  | Wild | Florida manatee (Trichechus manatus latirostris) | Crystal River, FL | Gut microbiome | (Merson et al., 2014) |
|  | Wild | Common bottlenose dolphin (Tursiops truncatus) | Southern California Bight, USA | Skin microbiome | (Russo et al., 2018) |
| ***Bdellovibrio*** |  |  |  |  |  |
|  | Wild | Common dolphins (Delphinus delphis), Striped dolphins (Stenella coeruleoalba) and Harbour porpoises (Phocoena phocoena) | Northern and Western Atlantic Iberian coast | Oral microbiome | (Soares-Castro et al., 2019) |
| ***Bifidobacterium*** |  |  |  |  |  |
|  | Wild | Southern right whale (Eubalaena australis) | Península Valdés, Argentina | Gut microbiome | (Maron et al., 2019) |
| ***Blautia*** |  |  |  |  |  |
|  | Wild | Australian sea lion (Neophoca cinerea) | Western and South Australia | Gut microbiome | (Delport et al. 2016) |
|  | Captive | Australian sea lion (Neophoca cinerea) | New South Wales and Gold Coast, Queensland, Australia | Gut microbiome | (Delport et al. 2016) |
|  | Wild | Common bottlenose dolphin (Tursiops truncatus) | Southern California Bight, USA | Skin microbiome | (Russo et al., 2018) |
| ***Brevibacterium*** |  |  |  |  |  |
|  | Wild | Southern Resident Killer Whale (Orcinus orca) | Salish Sea | Blow microbiome | (Raverty et al., 2017) |
| ***Burkholderia*** |  |  |  |  |  |
|  | Wild | Indo-Pacific bottlenose dolphins (Tursiops aduncus) | Shark Bay, Western Australia | Blow microbiome | (Nelson et al., 2019) |
|  | Wild | Southern Resident Killer Whale (Orcinus orca) | Salish Sea | Blow microbiome | (Raverty et al., 2017) |
|  | Wild | Humpback whale (Megaptera novaeangliae) | Queensland, Australia | Blow microbiome | (Vendl et al., 2019) |
| ***Butyricicoccus*** |  |  |  |  |  |
|  | Wild | East Asian finless porpoises (Neophocaena asiaeorientalis sunameri) | Penglai City, China | Gut microbiome | (Wan et al., 2018) |
| ***Butyrivibrio*** |  |  |  |  |  |
|  | Wild | Pygmy (Kogia breviceps) and Dwarf (Kogia sima) sperm whales | North Carolina | Gut microbiome | (Erwin et al., 2017) |
|  | Wild | Florida manatee (Trichechus manatus latirostris) | Crystal River, FL | Gut microbiome | (Merson et al., 2014) |
| ***Caloramator*** |  |  |  |  |  |
|  | Wild | Florida manatee (Trichechus manatus latirostris) | Crystal River, FL | Gut microbiome | (Merson et al., 2014) |
|  | Wild | Florida manatee (Trichechus manatus latirostris) | Crystal River, FL | Gut microbiome | (Merson et al., 2014) |
| ***Campylobacter*** |  |  |  |  |  |
|  | Wild | Common dolphins (Delphinus delphis), Striped dolphins (Stenella coeruleoalba) and Harbour porpoises (Phocoena phocoena) | Northern and Western Atlantic Iberian coast | Oral microbiome | (Soares-Castro et al., 2019) |
|  | Wild | Pygmy (Kogia breviceps) and Dwarf (Kogia sima) sperm whales | North Carolina | Gut microbiome | (Erwin et al., 2017) |
| ***Candidatus*** |  |  |  |  |  |
|  | Wild | Humpback whale (Megaptera novaeangliae) | Massachusetts and Washington, USA Vancouver, CAs | Blow microbiome | (Apprill et al., 2017) |
|  | Captive | Common bottlenose dolphin (Tursiops truncatus) and California sea lions (Zalophus californianus) | US Navy Marine Mammal Program, California, USA | Gut microbiome | (Bik et al., 2016) |
|  | Wild | Humpback whale (Megaptera novaeangliae) | Queensland, Australia | Blow microbiome | (Vendl et al., 2019) |
| ***Cardiobacterium*** |  |  |  |  |  |
|  | Wild | Humpback whale (Megaptera novaeangliae) | Southeast Alaska, Hawaiian Islands, Gulf of Maine, USA and American Samoa | Skin microbiome | (Apprill et al., 2014) |
|  | Wild | Humpback whale (Megaptera novaeangliae) | Massachusetts and Washington, USA Vancouver, CAs | Blow microbiome | (Apprill et al., 2017) |
|  | Captive | Common bottlenose dolphin (Tursiops truncatus) | US Navy Marine Mammal Program, California, USA | Oral microbiome | (Bik et al., 2016) |
|  | Wild | Common bottlenose dolphin (Tursiops truncatus) | Sarasota Bay, FL, USA | Oral microbiome | (Bik et al., 2016) |
| ***Caulobacter*** |  |  |  |  |  |
|  | Captive | Common bottlenose dolphins (Tursiops truncatus) | Antibes, France | Skin microbiome | (Chiarello et al., 2017) |
| ***Cellulosilyticum*** |  |  |  |  |  |
|  | Captive | Antillean manatee (Trichechus manatus manatus) | Ocean Expo Park, Okinawa, Japan | Gut microbiome | (Suzuki et al. 2019) |
|  | Wild | Florida manatee (Trichechus manatus latirostris) | Crystal River, FL | Gut microbiome | (Merson et al., 2014) |
| ***Cetobacterium*** |  |  |  |  |  |
|  | Wild | Common bottlenose dolphin (Tursiops truncatus) | Sarasota Bay, FL, USA | Gut microbiome | (Bik et al., 2016) |
|  | Captive | Common bottlenose dolphin (Tursiops truncatus) | US Navy Marine Mammal Program, California, USA | Gut microbiome | (Bik et al., 2016) |
|  | Wild | East Asian finless porpoises (Neophocaena asiaeorientalis sunameri) | Penglai City, China | Gut microbiome | (Wan et al., 2018) |
| ***Christensenella*** |  |  |  |  |  |
|  | Captive | Antillean manatee (Trichechus manatus manatus) | Ocean Expo Park, Okinawa, Japan | Gut microbiome | (Suzuki et al. 2019) |
| ***Chryseobacterium*** |  |  |  |  |  |
|  | Wild | Indo-Pacific bottlenose dolphins (Tursiops aduncus) | Shark Bay, Western Australia | Blow microbiome | (Nelson et al., 2019) |
|  | Wild | Common bottlenose dolphin (Tursiops truncatus) | Southern California Bight, USA | Skin microbiome | (Russo et al., 2018) |
| ***Citrobacter*** |  |  |  |  |  |
|  | Wild | Pygmy (Kogia breviceps) and Dwarf (Kogia sima) sperm whales | North Carolina | Gut microbiome | (Erwin et al., 2017) |
|  | Wild | Common bottlenose dolphin (Tursiops truncatus) | Southern California Bight, USA | Skin microbiome | (Russo et al., 2018) |
| ***Cloacibacterium*** |  |  |  |  |  |
|  | Wild | Humpback whale (Megaptera novaeangliae) | Western Antarctic Peninsula | Skin microbiome | (Bierlich et al., 2018) |
|  | Wild | Common bottlenose dolphin (Tursiops truncatus) | Southern California Bight, USA | Skin microbiome | (Russo et al., 2018) |
| ***Clostridium*** |  |  |  |  |  |
|  | Wild | Florida manatee (Trichechus manatus latirostris) | Crystal River, FL | Gut microbiome | (Merson et al., 2014) |
|  | Wild | Indo-Pacific bottlenose dolphins (Tursiops aduncus) | Shark Bay, Western Australia | Blow microbiome | (Nelson et al., 2019) |
|  | Wild | Pacific harbor seal (Phoca vitulina richardii) | Baja California, Mexico | Gut microbiome | (Pacheco-Sandoval et al., 2019) |
|  | Wild | Pygmy (Kogia breviceps) and Dwarf (Kogia sima) sperm whales | North Carolina | Gut microbiome | (Erwin et al., 2017) |
|  | Wild | Southern right whale (Eubalaena australis) | Península Valdés, Argentina | Gut microbiome | (Maron et al., 2019) |
|  | Wild | Humpback whale (Megaptera novaeangliae) | Western Antarctic Peninsula | Skin microbiome | (Bierlich et al., 2018) |
|  | Wild | East Asian finless porpoises (Neophocaena asiaeorientalis sunameri) | Penglai City, China | Gut microbiome | (Wan et al., 2018) |
|  | Captive | Antillean manatee (Trichechus manatus manatus) | Ocean Expo Park, Okinawa, Japan | Gut microbiome | (Suzuki et al. 2019) |
|  | Wild | Common dolphins (Delphinus delphis), Striped dolphins (Stenella coeruleoalba) and Harbour porpoises (Phocoena phocoena) | Northern and Western Atlantic Iberian coast | Oral microbiome | (Soares-Castro et al., 2019) |
|  | Captive | Antillean manatee (Trichechus manatus manatus) | Ocean Expo Park, Okinawa, Japan | Gut microbiome | (Suzuki et al. 2019) |
|  | Wild | Common bottlenose dolphin (Tursiops truncatus) | Southern California Bight, USA | Skin microbiome | (Russo et al., 2018) |
|  | Wild | Florida manatee (Trichechus manatus latirostris) | Crystal River, FL | Gut microbiome | (Merson et al., 2014) |
| ***Cobetia*** |  |  |  |  |  |
|  | Wild | Humpback whale (Megaptera novaeangliae) | Western Antarctic Peninsula | Skin microbiome | (Bierlich et al., 2018) |
| ***Colwellia*** |  |  |  |  |  |
|  | Wild | Humpback whale (Megaptera novaeangliae) | Southeast Alaska, Hawaiian Islands, Gulf of Maine, USA and American Samoa | Skin microbiome | (Apprill et al., 2014) |
| ***Comamonas*** |  |  |  |  |  |
|  | Wild | Common bottlenose dolphin (Tursiops truncatus) | Southern California Bight, USA | Skin microbiome | (Russo et al., 2018) |
| ***Coprococcus*** |  |  |  |  |  |
|  | Wild | Common bottlenose dolphin (Tursiops truncatus) | Southern California Bight, USA | Skin microbiome | (Russo et al., 2018) |
| ***Corynebacterium*** |  |  |  |  |  |
|  | Wild | Humpback whale (Megaptera novaeangliae) | Massachusetts and Washington, USA Vancouver, CAs | Blow microbiome | (Apprill et al., 2017) |
|  | Wild | Humpback whale (Megaptera novaeangliae) | Western Antarctic Peninsula | Skin microbiome | (Bierlich et al., 2018) |
|  | Wild | Southern right whale (Eubalaena australis) | Península Valdés, Argentina | Gut microbiome | (Maron et al., 2019) |
|  | Captive | Common bottlenose dolphin (Tursiops truncatus) | National Aquarium, Maryland, USA | Blow microbiome | (Nelson et al., 2019) |
|  | Wild | Indo-Pacific bottlenose dolphins (Tursiops aduncus) | Shark Bay, Western Australia | Blow microbiome | (Nelson et al., 2019) |
|  | Wild | Common bottlenose dolphin (Tursiops truncatus) | Southern California Bight, USA | Skin microbiome | (Russo et al., 2018) |
| ***Crocinitomix*** |  |  |  |  |  |
|  | Wild | Humpback whale (Megaptera novaeangliae) | Western Antarctic Peninsula | Skin microbiome | (Bierlich et al., 2018) |
| ***Cupriavidus*** |  |  |  |  |  |
|  | Wild | Killer whales (Orcinus orca) | North Pacific and Antartic | Skin microbiome | (Hooper et al., 2019) |
|  | Wild | Pacific harbor seal (Phoca vitulina richardii) | Baja California, Mexico | Gut microbiome | (Pacheco-Sandoval et al., 2019) |
| ***Curtobacterium pusillum*** |  |  |  |  |  |
|  | Wild | Southern Resident Killer Whale (Orcinus orca) | Salish Sea | Blow microbiome | (Raverty et al., 2017) |
| ***Cutibacterium*** |  |  |  |  |  |
|  | Wild | Killer whales (Orcinus orca) | North Pacific and Antartic | Skin microbiome | (Hooper et al., 2019) |
| ***Dechloromonas*** |  |  |  |  |  |
|  | Wild | Common bottlenose dolphin (Tursiops truncatus) | Southern California Bight, USA | Skin microbiome | (Russo et al., 2018) |
| ***Dermatophilus*** |  |  |  |  |  |
|  | Wild | Indo-Pacific bottlenose dolphins (Tursiops aduncus) | Shark Bay, Western Australia | Blow microbiome | (Nelson et al., 2019) |
| ***Dethiosulfovibrio*** |  |  |  |  |  |
|  | Wild | Harbour porpoises (Phocoena phocoena) | Northern and Western Atlantic Iberian coast | Oral microbiome | (Soares-Castro et al., 2019) |
| ***Diaphorobacter*** |  |  |  |  |  |
|  | Wild | Common bottlenose dolphin (Tursiops truncatus) | Southern California Bight, USA | Skin microbiome | (Russo et al., 2018) |
| ***Dielma*** |  |  |  |  |  |
|  | Wild | Humpback whale (Megaptera novaeangliae) | Massachusetts and Washington, USA Vancouver, CAs | Blow microbiome | (Apprill et al., 2017) |
| ***Dorea*** |  |  |  |  |  |
|  | Wild | Australian sea lion (Neophoca cinerea) | Western and South Australia | Gut microbiome | (Delport et al. 2016) |
|  | Captive | Australian sea lion (Neophoca cinerea) | New South Wales and Gold Coast, Queensland, Australia | Gut microbiome | (Delport et al. 2016) |
| ***Enhydrobacter*** |  |  |  |  |  |
|  | Captive | Killer whales (Orcinus orca) and Common bottlenose dolphins (Tursiops truncatus) | Antibes, France | Skin microbiome | (Chiarello et al., 2017) |
| ***Enterobacter*** |  |  |  |  |  |
|  | Wild | Common bottlenose dolphin (Tursiops truncatus) | Southern California Bight, USA | Skin microbiome | (Russo et al., 2018) |
| ***Epulopsicium*** |  |  |  |  |  |
|  | Wild | Florida manatee (Trichechus manatus latirostris) | Crystal River, FL | Gut microbiome | (Merson et al., 2014) |
| ***Erysipelothrix*** |  |  |  |  |  |
|  | Wild | Southern right whale (Eubalaena australis) | Península Valdés, Argentina | Gut microbiome | (Maron et al., 2019) |
|  | Wild | Southern right whale (Eubalaena australis) | Península Valdés, Argentina | Gut microbiome | (Maron et al., 2019) |
| ***Escherichia*** |  |  |  |  |  |
|  | Wild | Humpback whale (Megaptera novaeangliae) | Southeast Alaska, Hawaiian Islands, Gulf of Maine, USA and American Samoa | Skin microbiome | (Apprill et al., 2014) |
|  | Wild | Killer whales (Orcinus orca) | North Pacific and Antartic | Skin microbiome | (Hooper et al., 2019) |
|  | Wild | Southern right whale (Eubalaena australis) | Península Valdés, Argentina | Gut microbiome | (Maron et al., 2019) |
|  | Wild | Indo-Pacific bottlenose dolphins (Tursiops aduncus) | Shark Bay, Western Australia | Blow microbiome | (Nelson et al., 2019) |
| ***Eubacterium*** |  |  |  |  |  |
|  | Captive | Antillean manatee (Trichechus manatus manatus) | Ocean Expo Park, Okinawa, Japan | Gut microbiome | (Suzuki et al. 2019) |
|  | Captive | Antillean manatee (Trichechus manatus manatus) | Ocean Expo Park, Okinawa, Japan | Gut microbiome | (Suzuki et al. 2019) |
|  | Captive | Antillean manatee (Trichechus manatus manatus) | Ocean Expo Park, Okinawa, Japan | Gut microbiome | (Suzuki et al. 2019) |
|  | Wild | Florida manatee (Trichechus manatus latirostris) | Crystal River, FL | Gut microbiome | (Merson et al., 2014) |
| ***Faecalibacterium*** |  |  |  |  |  |
|  | Wild | Pacific harbor seal (Phoca vitulina richardii) | Baja California, Mexico | Gut microbiome | (Pacheco-Sandoval et al., 2019) |
|  | Wild | Common bottlenose dolphin (Tursiops truncatus) | Southern California Bight, USA | Skin microbiome | (Russo et al., 2018) |
|  | Wild | Pygmy (Kogia breviceps) and Dwarf (Kogia sima) sperm whales | North Carolina | Gut microbiome | (Erwin et al., 2017) |
| ***Fibrobacter*** |  |  |  |  |  |
|  | Wild | Common bottlenose dolphin (Tursiops truncatus) | Sarasota Bay, FL, USA | Oral microbiome | (Bik et al., 2016) |
|  | Captive | Common bottlenose dolphin (Tursiops truncatus) | US Navy Marine Mammal Program, California, USA | Oral microbiome | (Bik et al., 2016) |
| ***Flavobacterium*** |  |  |  |  |  |
|  | Wild | Humpback whale (Megaptera novaeangliae) | Western Antarctic Peninsula | Skin microbiome | (Bierlich et al., 2018) |
|  | Wild | Common bottlenose dolphin (Tursiops truncatus) | Southern California Bight, USA | Skin microbiome | (Russo et al., 2018) |
| ***Fusobacterium*** |  |  |  |  |  |
|  | Wild | Common bottlenose dolphin (Tursiops truncatus) | Sarasota Bay, FL, USA | Oral microbiome | (Bik et al., 2016) |
|  | Captive | Common bottlenose dolphin (Tursiops truncatus) | US Navy Marine Mammal Program, California, USA | Oral microbiome | (Bik et al., 2016) |
|  | Wild | Indo-Pacific bottlenose dolphins (Tursiops aduncus) | Shark Bay, Western Australia | Blow microbiome | (Nelson et al., 2019) |
|  | Wild | Pacific harbor seal (Phoca vitulina richardii) | Baja California, Mexico | Gut microbiome | (Pacheco-Sandoval et al., 2019) |
|  | Wild | Common dolphins (Delphinus delphis), Striped dolphins (Stenella coeruleoalba) and Harbour porpoises (Phocoena phocoena) | Northern and Western Atlantic Iberian coast | Oral microbiome | (Soares-Castro et al., 2019) |
|  | Wild | East Asian finless porpoises (Neophocaena asiaeorientalis sunameri) | Penglai City, China | Gut microbiome | (Wan et al., 2018) |
| ***Gardnerella*** |  |  |  |  |  |
|  | Captive | Killer whales (Orcinus orca) and Common bottlenose dolphins (Tursiops truncatus) | Antibes, France | Skin microbiome | (Chiarello et al., 2017) |
|  | Wild | Killer whales (Orcinus orca) | North Pacific and Antartic | Skin microbiome | (Hooper et al., 2019) |
| ***Gemella*** |  |  |  |  |  |
|  | Wild | Common dolphins (Delphinus delphis), Striped dolphins (Stenella coeruleoalba) and Harbour porpoises (Phocoena phocoena) | Northern and Western Atlantic Iberian coast | Oral microbiome | (Soares-Castro et al., 2019) |
| ***Geobacillus*** |  |  |  |  |  |
|  | Wild | Common bottlenose dolphin (Tursiops truncatus) | Southern California Bight, USA | Skin microbiome | (Russo et al., 2018) |
|  | Wild | Humpback whale (Megaptera novaeangliae) | Queensland, Australia | Blow microbiome | (Vendl et al., 2019) |
| ***Gracilibacteria*** |  |  |  |  |  |
|  | Wild | Humpback whale (Megaptera novaeangliae) | Western Antarctic Peninsula | Skin microbiome | (Bierlich et al., 2018) |
| ***Guggenheimella*** |  |  |  |  |  |
|  | Wild | Humpback whale (Megaptera novaeangliae) | Massachusetts and Washington, USA Vancouver, CAs | Blow microbiome | (Apprill et al., 2017) |
| ***Haemophilus*** |  |  |  |  |  |
|  | Wild | Common bottlenose dolphin (Tursiops truncatus) | Southern California Bight, USA | Skin microbiome | (Russo et al., 2018) |
| ***Halomonas*** |  |  |  |  |  |
|  | Wild | Southern Resident Killer Whale (Orcinus orca) | Salish Sea | Blow microbiome | (Raverty et al., 2017) |
|  | Wild | Southern Resident Killer Whale (Orcinus orca) | Salish Sea | Blow microbiome | (Raverty et al., 2017) |
| ***Helcococcus*** |  |  |  |  |  |
|  | Wild | Humpback whale (Megaptera novaeangliae) | Massachusetts and Washington, USA Vancouver, CAs | Blow microbiome | (Apprill et al., 2017) |
| ***Helicobacter*** |  |  |  |  |  |
|  | Captive | Common bottlenose dolphin (Tursiops truncatus) and California sea lions (Zalophus californianus) | US Navy Marine Mammal Program, California, USA | Gut microbiome | (Bik et al., 2016) |
|  | Wild | Southern right whale (Eubalaena australis) | Península Valdés, Argentina | Gut microbiome | (Maron et al., 2019) |
|  | Wild | East Asian finless porpoises (Neophocaena asiaeorientalis sunameri) | Penglai City, China | Gut microbiome | (Wan et al., 2018) |
| ***Helocococcus*** |  |  |  |  |  |
|  | Captive | Common bottlenose dolphin (Tursiops truncatus) | National Aquarium, Maryland, USA | Blow microbiome | (Nelson et al., 2019) |
|  | Wild | Indo-Pacific bottlenose dolphins (Tursiops aduncus) | Shark Bay, Western Australia | Blow microbiome | (Nelson et al., 2019) |
| ***Intestinimonas*** |  |  |  |  |  |
|  | Captive | Antillean manatee (Trichechus manatus manatus) | Ocean Expo Park, Okinawa, Japan | Gut microbiome | (Suzuki et al. 2019) |
| ***Kocuria*** |  |  |  |  |  |
|  | Wild | Southern Resident Killer Whale (Orcinus orca) | Salish Sea | Blow microbiome | (Raverty et al., 2017) |
| ***Leucobacter*** |  |  |  |  |  |
|  | Wild | Humpback whale (Megaptera novaeangliae) | Massachusetts and Washington, USA Vancouver, CAs | Blow microbiome | (Apprill et al., 2017) |
| ***Marinicella*** |  |  |  |  |  |
|  | Wild | Harbour porpoises (Phocoena phocoena) | Northern and Western Atlantic Iberian coast | Oral microbiome | (Soares-Castro et al., 2019) |
| ***Maritimimonas*** |  |  |  |  |  |
|  | Wild | Common dolphins (Delphinus delphis), Striped dolphins (Stenella coeruleoalba) and Harbour porpoises (Phocoena phocoena) | Northern and Western Atlantic Iberian coast | Oral microbiome | (Soares-Castro et al., 2019) |
| ***Methylobacterium*** |  |  |  |  |  |
|  | Wild | Indo-Pacific bottlenose dolphins (Tursiops aduncus) | Shark Bay, Western Australia | Blow microbiome | (Nelson et al., 2019) |
|  | Wild | Common bottlenose dolphin (Tursiops truncatus) | Southern California Bight, USA | Skin microbiome | (Russo et al., 2018) |
| ***Microbacterium*** |  |  |  |  |  |
|  | Wild | Southern Resident Killer Whale (Orcinus orca) | Salish Sea | Blow microbiome | (Raverty et al., 2017) |
| ***Micromonospora auratinigra*** |  |  |  |  |  |
|  | Wild | Harbor porpoise (Phocoena phocoena) | Santa Cruz, CA, USA | Gut microbiome | (Ochoa et al., 2018) |
| ***Microvirga*** |  |  |  |  |  |
|  | Captive | Killer whales (Orcinus orca) | Antibes, France | Skin microbiome | (Chiarello et al., 2017) |
| ***Moraxella*** |  |  |  |  |  |
|  | Wild | Humpback whale (Megaptera novaeangliae) | Massachusetts and Washington, USA Vancouver, CAs | Blow microbiome | (Apprill et al., 2017) |
| ***Morganella*** |  |  |  |  |  |
|  | Wild | East Asian finless porpoises (Neophocaena asiaeorientalis sunameri) | Penglai City, China | Gut microbiome | (Wan et al., 2018) |
| ***Mycobacterium arupense*** |  |  |  |  |  |
|  | Wild | Pygmy (Kogia breviceps) and Dwarf (Kogia sima) sperm whales | North Carolina | Gut microbiome | (Erwin et al., 2017) |
| ***Mycoplasma*** |  |  |  |  |  |
|  | Captive | Common bottlenose dolphin (Tursiops truncatus) | National Aquarium, Maryland, USA | Blow microbiome | (Nelson et al., 2019) |
|  | Wild | Indo-Pacific bottlenose dolphins (Tursiops aduncus) | Shark Bay, Western Australia | Blow microbiome | (Nelson et al., 2019) |
|  | Wild | Southern right whale (Eubalaena australis) | Península Valdés, Argentina | Gut microbiome | (Maron et al., 2019) |
| ***Novosphingobium*** |  |  |  |  |  |
|  | Wild | Humpback whale (Megaptera novaeangliae) | Southeast Alaska, Hawaiian Islands, Gulf of Maine, USA and American Samoa | Skin microbiome | (Apprill et al., 2014) |
|  | Wild | Indo-Pacific bottlenose dolphins (Tursiops aduncus) | Shark Bay, Western Australia | Blow microbiome | (Nelson et al., 2019) |
| ***Oceanivirga*** |  |  |  |  |  |
|  | Wild | Humpback whale (Megaptera novaeangliae) | Massachusetts and Washington, USA Vancouver, CAs | Blow microbiome | (Apprill et al., 2017) |
|  | Wild | Common dolphins (Delphinus delphis), Striped dolphins (Stenella coeruleoalba) and Harbour porpoises (Phocoena phocoena) | Northern and Western Atlantic Iberian coast | Oral microbiome | (Soares-Castro et al., 2019) |
| ***Oscillospira*** |  |  |  |  |  |
|  | Wild | Pygmy (Kogia breviceps) and Dwarf (Kogia sima) sperm whales | North Carolina | Gut microbiome | (Erwin et al., 2017) |
|  | Wild | Pacific harbor seal (Phoca vitulina richardii) | Baja California, Mexico | Gut microbiome | (Pacheco-Sandoval et al., 2019) |
|  | Wild | Southern right whale (Eubalaena australis) | Península Valdés, Argentina | Gut microbiome | (Maron et al., 2019) |
| ***Owenweeksia*** |  |  |  |  |  |
|  | Wild | Humpback whale (Megaptera novaeangliae) | Western Antarctic Peninsula | Skin microbiome | (Bierlich et al., 2018) |
| ***Paracoccus*** |  |  |  |  |  |
|  | Captive | Killer whales (Orcinus orca) and Common bottlenose dolphins (Tursiops truncatus) | Antibes, France | Skin microbiome | (Chiarello et al., 2017) |
|  | Wild | Common bottlenose dolphin (Tursiops truncatus) | Southern California Bight, USA | Skin microbiome | (Russo et al., 2018) |
| ***Pasteurella*** |  |  |  |  |  |
|  | Wild | Southern right whale (Eubalaena australis) | Península Valdés, Argentina | Gut microbiome | (Maron et al., 2019) |
| ***Pedomicrobium*** |  |  |  |  |  |
|  | Captive | Common bottlenose dolphin (Tursiops truncatus) | National Aquarium, Maryland, USA | Blow microbiome | (Nelson et al., 2019) |
|  | Wild | Indo-Pacific bottlenose dolphins (Tursiops aduncus) | Shark Bay, Western Australia | Blow microbiome | (Nelson et al., 2019) |
| ***Peptococcus*** |  |  |  |  |  |
|  | Wild | Australian sea lion (Neophoca cinerea) | Western and South Australia | Gut microbiome | (Delport et al. 2016) |
|  | Captive | Australian sea lion (Neophoca cinerea) | New South Wales and Gold Coast, Queensland, Australia | Gut microbiome | (Delport et al. 2016) |
| ***Peptostreptococcus*** |  |  |  |  |  |
|  | Wild | Common dolphins (Delphinus delphis), Striped dolphins (Stenella coeruleoalba) and Harbour porpoises (Phocoena phocoena) | Northern and Western Atlantic Iberian coast | Oral microbiome | (Soares-Castro et al., 2019) |
| ***Phascolarctobacterium*** |  |  |  |  |  |
|  | Wild | Pacific harbor seal (Phoca vitulina richardii) | Baja California, Mexico | Gut microbiome | (Pacheco-Sandoval et al., 2019) |
| ***Phenylobacterium*** |  |  |  |  |  |
|  | Captive | Common bottlenose dolphin (Tursiops truncatus) | National Aquarium, Maryland, USA | Blow microbiome | (Nelson et al., 2019) |
| ***Phocoenobacter*** |  |  |  |  |  |
|  | Wild | Indo-Pacific bottlenose dolphins (Tursiops aduncus) | Shark Bay, Western Australia | Blow microbiome | (Nelson et al., 2019) |
|  | Wild | Common dolphins (Delphinus delphis), Striped dolphins (Stenella coeruleoalba) and Harbour porpoises (Phocoena phocoena) | Northern and Western Atlantic Iberian coast | Oral microbiome | (Soares-Castro et al., 2019) |
| ***Photobacterium*** |  |  |  |  |  |
|  | Wild | East Asian finless porpoises (Neophocaena asiaeorientalis sunameri) | Penglai City, China | Gut microbiome | (Wan et al., 2018) |
|  | Wild | Pacific harbor seal (Phoca vitulina richardii) | Baja California, Mexico | Gut microbiome | (Pacheco-Sandoval et al., 2019) |
| ***Polaribacter*** |  |  |  |  |  |
|  | Wild | Humpback whale (Megaptera novaeangliae) | Western Antarctic Peninsula | Skin microbiome | (Bierlich et al., 2018) |
| ***Porphyromonas*** |  |  |  |  |  |
|  | Wild | Humpback whale (Megaptera novaeangliae) | Massachusetts and Washington, USA Vancouver, CAs | Blow microbiome | (Apprill et al., 2017) |
|  | Wild | Common dolphins (Delphinus delphis), Striped dolphins (Stenella coeruleoalba) and Harbour porpoises (Phocoena phocoena) | Northern and Western Atlantic Iberian coast | Oral microbiome | (Soares-Castro et al., 2019) |
| ***Propionibacterium*** |  |  |  |  |  |
|  | Wild | Common bottlenose dolphin (Tursiops truncatus) | Southern California Bight, USA | Skin microbiome | (Russo et al., 2018) |
|  | Wild | Humpback whale (Megaptera novaeangliae) | Queensland, Australia | Blow microbiome | (Vendl et al., 2019) |
| ***Pseudoalteromonas*** |  |  |  |  |  |
|  | Wild | Humpback whale (Megaptera novaeangliae) | Western Antarctic Peninsula | Skin microbiome | (Bierlich et al., 2018) |
|  | Wild | Killer whales (Orcinus orca) | North Pacific and Antartic | Skin microbiome | (Hooper et al., 2019) |
| ***Pseudomonas*** |  |  |  |  |  |
|  | Wild | Humpback whale (Megaptera novaeangliae) | Western Antarctic Peninsula | Skin microbiome | (Bierlich et al., 2018) |
|  | Wild | Indo-Pacific bottlenose dolphins (Tursiops aduncus) | Shark Bay, Western Australia | Blow microbiome | (Nelson et al., 2019) |
|  | Wild | Southern Resident Killer Whale (Orcinus orca) | Salish Sea | Blow microbiome | (Raverty et al., 2017) |
|  | Wild | Common bottlenose dolphin (Tursiops truncatus) | Southern California Bight, USA | Skin microbiome | (Russo et al., 2018) |
|  | Wild | East Asian finless porpoises (Neophocaena asiaeorientalis sunameri) | Penglai City, China | Gut microbiome | (Wan et al., 2018) |
|  | Wild | Southern Resident Killer Whale (Orcinus orca) | Salish Sea | Blow microbiome | (Raverty et al., 2017) |
| ***Psychrobacter*** |  |  |  |  |  |
|  | Wild | Humpback whale (Megaptera novaeangliae) | Massachusetts and Washington, USA Vancouver, CAs | Blow microbiome | (Apprill et al., 2017) |
|  | Captive | Killer whales (Orcinus orca) and Common bottlenose dolphins (Tursiops truncatus) | Antibes, France | Skin microbiome | (Chiarello et al., 2017) |
|  | Wild | Humpback whale (Megaptera novaeangliae) | Southeast Alaska, Hawaiian Islands, Gulf of Maine, USA and American Samoa | Skin microbiome | (Apprill et al., 2014) |
|  | Wild | Humpback whale (Megaptera novaeangliae) | Western Antarctic Peninsula | Skin microbiome | (Bierlich et al., 2018) |
|  | Wild | Southern Resident Killer Whale (Orcinus orca) | Salish Sea | Blow microbiome | (Raverty et al., 2017) |
|  | Wild | Common bottlenose dolphin (Tursiops truncatus) | Southern California Bight, USA | Skin microbiome | (Russo et al., 2018) |
| ***Psychromonas arctica*** |  |  |  |  |  |
|  | Wild | Southern Resident Killer Whale (Orcinus orca) | Salish Sea | Blow microbiome | (Raverty et al., 2017) |
| ***Ralstonia*** |  |  |  |  |  |
|  | Wild | Common dolphins (Delphinus delphis), Striped dolphins (Stenella coeruleoalba) and Harbour porpoises (Phocoena phocoena) | Northern and Western Atlantic Iberian coast | Oral microbiome | (Soares-Castro et al., 2019) |
| ***Rheinheimera*** |  |  |  |  |  |
|  | Wild | Southern Resident Killer Whale (Orcinus orca) | Salish Sea | Blow microbiome | (Raverty et al., 2017) |
| ***Rhodovulum*** |  |  |  |  |  |
|  | Wild | Humpback whale (Megaptera novaeangliae) | Massachusetts and Washington, USA Vancouver, CAs | Blow microbiome | (Apprill et al., 2017) |
| ***Roseburia*** |  |  |  |  |  |
|  | Wild | Common bottlenose dolphin (Tursiops truncatus) | Southern California Bight, USA | Skin microbiome | (Russo et al., 2018) |
| ***Rothia*** |  |  |  |  |  |
|  | Wild | Southern Resident Killer Whale (Orcinus orca) | Salish Sea | Blow microbiome | (Raverty et al., 2017) |
| ***Rubellimicrobium*** |  |  |  |  |  |
|  | Captive | Killer whales (Orcinus orca) | Antibes, France | Skin microbiome | (Chiarello et al., 2017) |
| ***Ruegeria*** |  |  |  |  |  |
|  | Captive | Killer whales (Orcinus orca) | Antibes, France | Skin microbiome | (Chiarello et al., 2017) |
| ***Ruminococcus*** |  |  |  |  |  |
|  | Wild | Florida manatee (Trichechus manatus latirostris) | Crystal River, FL | Gut microbiome | (Merson et al., 2014) |
| ***Salmonella enterica*** |  |  |  |  |  |
|  | Wild | Southern Resident Killer Whale (Orcinus orca) | Salish Sea | Blow microbiome | (Raverty et al., 2017) |
| ***Sedimentibacter*** |  |  |  |  |  |
|  | Wild | Florida manatee (Trichechus manatus latirostris) | Crystal River, FL | Gut microbiome | (Merson et al., 2014) |
| ***Serratia*** |  |  |  |  |  |
|  | Wild | Common bottlenose dolphin (Tursiops truncatus) | Southern California Bight, USA | Skin microbiome | (Russo et al., 2018) |
| ***Slackia*** |  |  |  |  |  |
|  | Wild | Pacific harbor seal (Phoca vitulina richardii) | Baja California, Mexico | Gut microbiome | (Pacheco-Sandoval et al., 2019) |
| ***Sphingobacterium*** |  |  |  |  |  |
|  | Wild | Common bottlenose dolphin (Tursiops truncatus) | Southern California Bight, USA | Skin microbiome | (Russo et al., 2018) |
| ***Sphingomonas*** |  |  |  |  |  |
|  | Captive | Killer whales (Orcinus orca) and Common bottlenose dolphins (Tursiops truncatus) | Antibes, France | Skin microbiome | (Chiarello et al., 2017) |
|  | Wild | Indo-Pacific bottlenose dolphins (Tursiops aduncus) | Shark Bay, Western Australia | Blow microbiome | (Nelson et al., 2019) |
|  | Wild | Common bottlenose dolphin (Tursiops truncatus) | Southern California Bight, USA | Skin microbiome | (Russo et al., 2018) |
| ***Sporosarcina*** |  |  |  |  |  |
|  | Wild | Southern Resident Killer Whale (Orcinus orca) | Salish Sea | Blow microbiome | (Raverty et al., 2017) |
|  | Wild | Southern Resident Killer Whale (Orcinus orca) | Salish Sea | Blow microbiome | (Raverty et al., 2017) |
| ***Staphylococcus*** |  |  |  |  |  |
|  | Wild | Humpback whale (Megaptera novaeangliae) | Western Antarctic Peninsula | Skin microbiome | (Bierlich et al., 2018) |
|  | Captive | Killer whales (Orcinus orca) and Common bottlenose dolphins (Tursiops truncatus) | Antibes, France | Skin microbiome | (Chiarello et al., 2017) |
|  | Wild | Killer whales (Orcinus orca) | North Pacific and Antartic | Skin microbiome | (Hooper et al., 2019) |
|  | Wild | Indo-Pacific bottlenose dolphins (Tursiops aduncus) | Shark Bay, Western Australia | Blow microbiome | (Nelson et al., 2019) |
|  | Wild | Southern Resident Killer Whale (Orcinus orca) | Salish Sea | Blow microbiome | (Raverty et al., 2017) |
|  | Wild | Southern Resident Killer Whale (Orcinus orca) | Salish Sea | Blow microbiome | (Raverty et al., 2017) |
|  | Wild | Southern Resident Killer Whale (Orcinus orca) | Salish Sea | Blow microbiome | (Raverty et al., 2017) |
|  | Wild | Southern Resident Killer Whale (Orcinus orca) | Salish Sea | Blow microbiome | (Raverty et al., 2017) |
|  | Wild | Southern Resident Killer Whale (Orcinus orca) | Salish Sea | Blow microbiome | (Raverty et al., 2017) |
|  | Wild | Southern Resident Killer Whale (Orcinus orca) | Salish Sea | Blow microbiome | (Raverty et al., 2017) |
|  | Wild | Southern Resident Killer Whale (Orcinus orca) | Salish Sea | Blow microbiome | (Raverty et al., 2017) |
| ***Stenotrophomonas*** |  |  |  |  |  |
|  | Wild | Indo-Pacific bottlenose dolphins (Tursiops aduncus) | Shark Bay, Western Australia | Blow microbiome | (Nelson et al., 2019) |
|  | Wild | Southern Resident Killer Whale (Orcinus orca) | Salish Sea | Blow microbiome | (Raverty et al., 2017) |
| ***Streptobacillus*** |  |  |  |  |  |
|  | Wild | Indo-Pacific bottlenose dolphins (Tursiops aduncus) | Shark Bay, Western Australia | Blow microbiome | (Nelson et al., 2019) |
| ***Streptococcus*** |  |  |  |  |  |
|  | Wild | Humpback whale (Megaptera novaeangliae) | Western Antarctic Peninsula | Skin microbiome | (Bierlich et al., 2018) |
|  | Wild | Killer whales (Orcinus orca) | North Pacific and Antartic | Skin microbiome | (Hooper et al., 2019) |
|  | Wild | Indo-Pacific bottlenose dolphins (Tursiops aduncus) | Shark Bay, Western Australia | Blow microbiome | (Nelson et al., 2019) |
|  | Wild | Common bottlenose dolphin (Tursiops truncatus) | Southern California Bight, USA | Skin microbiome | (Russo et al., 2018) |
|  | Wild | Southern right whale (Eubalaena australis) | Península Valdés, Argentina | Gut microbiome | (Maron et al., 2019) |
| ***Streptomyces*** |  |  |  |  |  |
|  | Wild | Southern Resident Killer Whale (Orcinus orca) | Salish Sea | Blow microbiome | (Raverty et al., 2017) |
| ***Sutterella*** |  |  |  |  |  |
|  | Wild | Australian sea lion (Neophoca cinerea) | Western and South Australia | Gut microbiome | (Delport et al. 2016) |
|  | Captive | Australian sea lion (Neophoca cinerea) | New South Wales and Gold Coast, Queensland, Australia | Gut microbiome | (Delport et al. 2016) |
| ***Suttonella*** |  |  |  |  |  |
|  | Wild | North Pacific Humpback whale (Megaptera novaeangliae) | Hawaiian Islands, USA | Skin microbiome | (Apprill et al., 2011) |
|  | Wild | Humpback whale (Megaptera novaeangliae) | Southeast Alaska, Hawaiian Islands, Gulf of Maine, USA and American Samoa | Skin microbiome | (Apprill et al., 2014) |
| ***Tenacibaculum*** |  |  |  |  |  |
|  | Wild | Humpback whale (Megaptera novaeangliae) | Western Antarctic Peninsula | Skin microbiome | (Bierlich et al., 2018) |
|  | Wild | Killer whales (Orcinus orca) | North Pacific and Antartic | Skin microbiome | (Hooper et al., 2019) |
|  | Captive | Common bottlenose dolphin (Tursiops truncatus) | National Aquarium, Maryland, USA | Blow microbiome | (Nelson et al., 2019) |
|  | Wild | Indo-Pacific bottlenose dolphins (Tursiops aduncus) | Shark Bay, Western Australia | Blow microbiome | (Nelson et al., 2019) |
|  | Wild | North Pacific Humpback whale (Megaptera novaeangliae) | Hawaiian Islands, USA | Skin microbiome | (Apprill et al., 2011) |
|  | Wild | Humpback whale (Megaptera novaeangliae) | Southeast Alaska, Hawaiian Islands, Gulf of Maine, USA and American Samoa | Skin microbiome | (Apprill et al., 2014) |
|  | Wild | Humpback whale (Megaptera novaeangliae) | Massachusetts and Washington, USA Vancouver, CAs | Blow microbiome | (Apprill et al., 2017) |
| ***Terrisporobacter*** |  |  |  |  |  |
|  | Captive | Antillean manatee (Trichechus manatus manatus) | Ocean Expo Park, Okinawa, Japan | Gut microbiome | (Suzuki et al. 2019) |
| ***Ureaplasma*** |  |  |  |  |  |
|  | Wild | East Asian finless porpoises (Neophocaena asiaeorientalis sunameri) | Penglai City, China | Gut microbiome | (Wan et al., 2018) |
| ***Vibrio*** |  |  |  |  |  |
|  | Wild | Pacific harbor seal (Phoca vitulina richardii) | Baja California, Mexico | Gut microbiome | (Pacheco-Sandoval et al., 2019) |
|  | Wild | East Asian finless porpoises (Neophocaena asiaeorientalis sunameri) | Penglai City, China | Gut microbiome | (Wan et al., 2018) |
|  | Wild | Southern Resident Killer Whale (Orcinus orca) | Salish Sea | Blow microbiome | (Raverty et al., 2017) |
|  | Wild | Southern Resident Killer Whale (Orcinus orca) | Salish Sea | Blow microbiome | (Raverty et al., 2017) |
|  | Wild | Southern Resident Killer Whale (Orcinus orca) | Salish Sea | Blow microbiome | (Raverty et al., 2017) |
| ***Zimmermannella*** |  |  |  |  |  |
|  | Wild | Humpback whale (Megaptera novaeangliae) | Massachusetts and Washington, USA Vancouver, CAs | Blow microbiome | (Apprill et al., 2017) |
